# Comparative genome and transcriptome analysis reveals distinctive surface characteristics and unique physiological potentials of *Pseudomonas aeruginosa* ATCC 27853

**DOI:** 10.1186/s12864-017-3842-z

**Published:** 2017-06-12

**Authors:** Huiluo Cao, Yong Lai, Salim Bougouffa, Zeling Xu, Aixin Yan

**Affiliations:** 1 School of Biological Sciences, The University of Hong Kong, Pokfulam Road, Hong Kong, China; 2 Computational Bioscience Research Center (CBRC), King Abdullah University of Science and Technology (KAUST), Thuwal, Saudi Arabia; 3 Present address: Interdisciplinary research group in Infectious Diseases, The Singapore-MIT Alliance for Research and Technology (SMART), Singapore, Singapore

**Keywords:** *Pseudomonas Aeruginosa*, Prophage, Genomic islands, Comparative transcriptome analysis, RNA-seq, Secretion system, Colony morphology

## Abstract

**Background:**

*Pseudomonas aeruginosa* ATCC 27853 was isolated from a hospital blood specimen in 1971 and has been widely used as a model strain to survey antibiotics susceptibilities, biofilm development, and metabolic activities of *Pseudomonas* spp.. Although four draft genomes of *P. aeruginosa* ATCC 27853 have been sequenced, the complete genome of this strain is still lacking, hindering a comprehensive understanding of its physiology and functional genome.

**Results:**

Here we sequenced and assembled the complete genome of *P. aeruginosa* ATCC 27853 using the Pacific Biosciences SMRT (PacBio) technology and Illumina sequencing platform. We found that accessory genes of ATCC 27853 including prophages and genomic islands (GIs) mainly contribute to the difference between *P. aeruginosa* ATCC 27853 and other *P. aeruginosa* strains. Seven prophages were identified within the genome of *P. aeruginosa* ATCC 27853. Of the predicted 25 GIs, three contain genes that encode monoxoygenases, dioxygenases and hydrolases that could be involved in the metabolism of aromatic compounds. Surveying virulence-related genes revealed that a series of genes that encode the B-band O-antigen of LPS are lacking in ATCC 27853. Distinctive SNPs in genes of cellular adhesion proteins such as type IV pili and flagella biosynthesis were also observed in this strain. Colony morphology analysis confirmed an enhanced biofilm formation capability of ATCC 27853 on solid agar surface compared to *Pseudomonas aeruginosa* PAO1. We then performed transcriptome analysis of ATCC 27853 and PAO1 using RNA-seq and compared the expression of orthologous genes to understand the functional genome and the genomic details underlying the distinctive colony morphogenesis. These analyses revealed an increased expression of genes involved in cellular adhesion and biofilm maturation such as type IV pili, exopolysaccharide and electron transport chain components in ATCC 27853 compared with PAO1. In addition, distinctive expression profiles of the virulence genes *lecA*, *lasB*, quorum sensing regulators LasI/R, and the type I, III and VI secretion systems were observed in the two strains.

**Conclusions:**

The complete genome sequence of *P. aeruginosa* ATCC 27853 reveals the comprehensive genetic background of the strain, and provides genetic basis for several interesting findings about the functions of surface associated proteins, prophages, and genomic islands. Comparative transcriptome analysis of *P. aeruginosa* ATCC 27853 and PAO1 revealed several classes of differentially expressed genes in the two strains, underlying the genetic and molecular details of several known and yet to be explored morphological and physiological potentials of *P. aeruginosa* ATCC 27853.

**Electronic supplementary material:**

The online version of this article (doi:10.1186/s12864-017-3842-z) contains supplementary material, which is available to authorized users.

## Background

*Pseudomonas aeruginosa* is a gram-negative, broad-host range, opportunistic pathogen found in diverse ecological niches. It is a frequent cause of many human infectious diseases including keratitis, burn infections, urinary tract infections (UTIs), sepsis, as well as acute and chronic infections of human airways. To understand the adaptation and pathogenesis of the bacterium, comprehensive investigations of the genomes and transcriptomes of *P. aeruginosa* strains from various sources are necessary.

Typical *P. aeruginosa* strains have a large genome size of 6–7 Mb encoding around 6000 genes contributing to the versatility of the species [1, 2]. The architecture of *P. aeruginosa* genomes exhibit a mosaic pattern composed of a core genome (5316 core genes) and a series of accessory genes inserted sporadically, including prophages, plasmids and islets [3]. Accessory genes could be acquired by horizontal gene transfer from various sources and they often contribute to the unique physiology, pathogenesis, or transmission capacity of the corresponding strains as has been demonstrated in several *P. aeruginosa* isolates [4, 5]. Although over one thousand genomes (deposited in NCBI GenBank) of *P. aeruginosa* have been sequenced, only 58 (as of May 2016) complete genomes are available, limiting a comprehensive understanding of this important group of opportunistic pathogens.

*P. aeruginosa* ATCC 27853 is commonly used in biomedical research and was initially isolated from a blood specimen in the Peter Bent Brigham Hospital in 1971 (Boston, USA) [6]. ATCC 27853 has been widely used as a model strain to survey antibiotics susceptibilities since 1978 [7, 8]. So far, four draft genomes of *P. aeruginosa* ATCC 27853 have been sequenced [9–12], but the complete genome of the strain is still lacking, hindering the understanding of its full physiological potentials.

In the present study, we sequenced and assembled the complete genome of *P. aeruginosa* ATCC 27853 using both PacBio’s SMRT and Illumina platforms. We then compared it with the complete genomes of two frequently used *P. aeruginosa* laboratory strains, *P. aeruginosa* PAO1 and *P. aeruginosa* PA14, to reveal distinct features of the ATCC 27853 genome. To advance our understanding of the physiology of the strain, specifically its morphogenesis, we performed comparative transcriptome analysis on ATCC 27853 and PAO1. These analyses revealed the presence of a large number (seven) of prophages in its genome and several unique physiological features of ATCC 27853, implying the striking ability of the strain to adapt to a variety of environmental niches and stresses.

## Results

### General features of the genome of *P. aeruginosa* ATCC27853

A total of 1.296 Gb raw data were produced by the PacBio platform. The error correction step produced 146,425 reads with an average length of 7564 bp and a maximum length of 39,699 bp. Corrected reads were assembled de novo, the contig was then polished and circularized using the SMRT Analysis pipeline to produce a single 6.833 Mb contig with 158× coverage. The assembly was also validated by mapping Illumina-generated reads. The GC content of the genome was 66.12%, which is comparable to other genomes within the *P. aeruginosa* species (Additional file 1: Table S1). A total of 6366 genes were predicted. Twelve rRNA genes, 66 tRNA genes and 215 tandem repeats were identified (Table 1).

### Phylogenetic relationship of the ATCC 27853 with other *P. aeruginosa* strains based on SNPs from all complete genomes

**Fig. 1:**
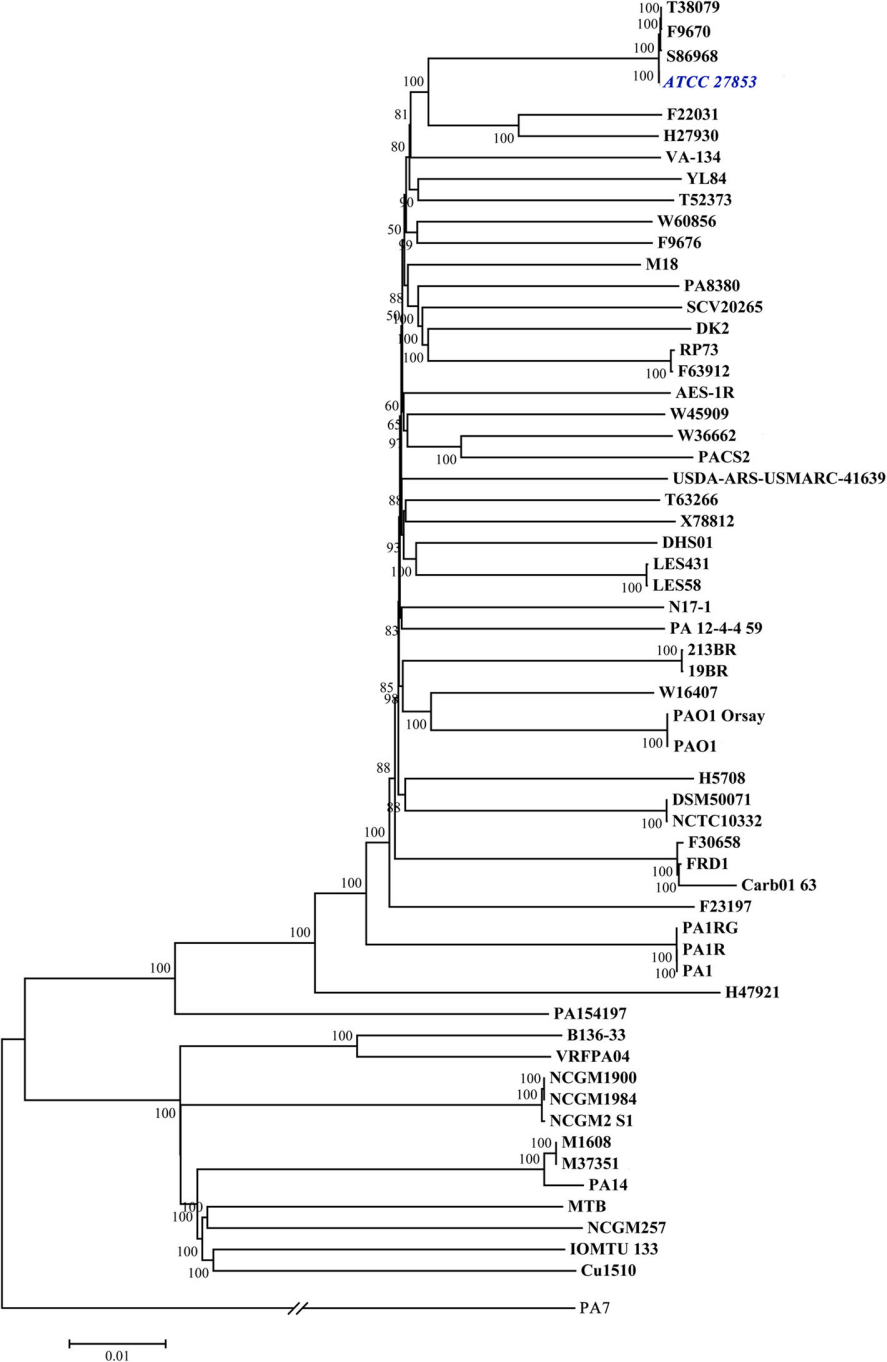
Phylogenetic relationships of the currently available 59 complete genomes of *Pseudomonas aeruginosa* constructed based on the SNPs identified using Harvest with 100 bootstrap and maximum likelihood (ML) criterion in MEGA software. *P. aeruginosa* ATCC 27853 is highlighted in *blue* and italic style. The denotation of the strain is listed in the Additional file 1: Table S1

Since the 16S rRNA genes in the different strains of the *P. aeruginosa* species exhibit high similarity (>99%, data not shown) with low discriminating capability, single nucleotide polymorphisms (SNPs) were used to construct the phylogenetic relationship between ATCC 27853 and published strains. Using Harvest [13], we collected 269,561 SNPs from the complete genomes included. We generated the phylogenetic tree in MEGA [14] based on the maximum likelihood (ML) algorithm. It became apparent that *P. aeruginosa* ATCC 27853 is closely related to *P. aeruginosa* T38079, *P. aeruginosa* F9670 and *P. aeruginosa* S86968, all of which are clinical isolates (Fig. 1, Additional file 1: Table S1).

### COG comparison

**Fig. 2:**
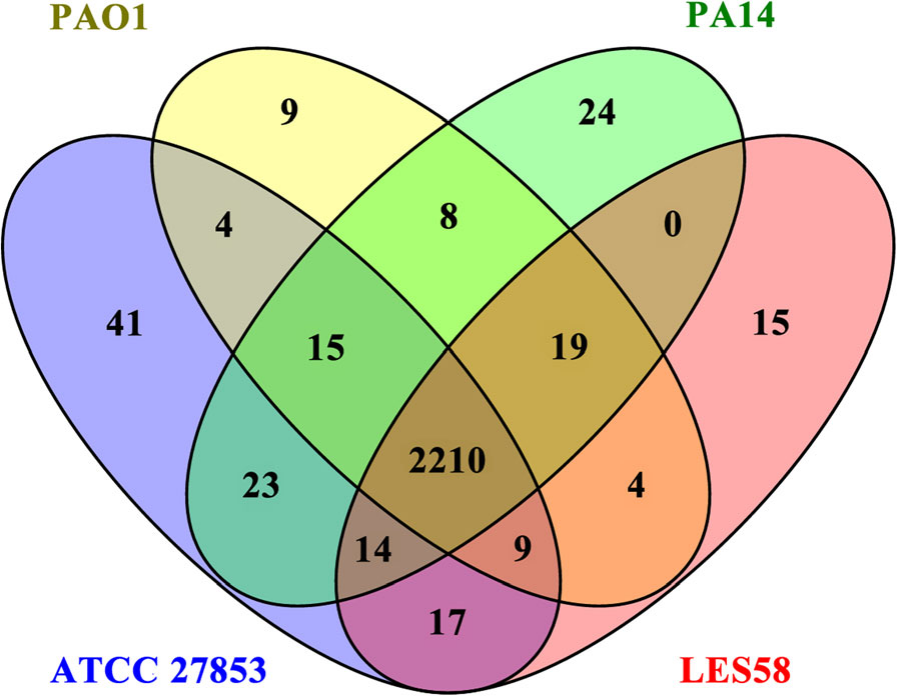
Venn diagram showing the number of shared and exclusive genes among four *P. aeruginosa* strains: *P. aeruginosa* ATCC 27853, *P. aeruginosa* PAO1, *P. aeruginosa* PA14, *P. aeruginosa* LESB58. The number of unique genes, those shared among two, three and all four strains of ATCC 27853, PAO1, PA14 and LESB58 strains based on the COG gene annotations are shown

We compared Clusters of Orthologous Groups (COG) annotations of *P. aeruginosa* ATCC 27853 with those of *P. aeruginosa* PAO1, *P. aeruginosa* PA14 and *P. aeruginosa* LESB58 (an epidemic strain with known prophage functions) (Fig. 2 and Table 2). A total of 41 COGs are exclusively present in *P. aeruginosa* ATCC 27853 (Fig. 2 and Table 2), a much higher number if compared with the unique COGs in the other three genomes (Fig. 2). Most of these COGs are phage and plasmid proteins, consistent with the high number of prophages (seven) identified in *P. aeruginosa* ATCC 27853 (below). In addition, 58 COGs in *P. aeruginosa* ATCC 27853 are absent in *P. aeruginosa* PAO1. Nineteen of these genes have uncharacterized functions or with only hypothetical functions (Table 2). Several site-specific DNA methylase (COG0270 and COG0338) are also present in the list (Table 2).

### Genomic islands

A total of 25 genomic islands (GIs) were identified in the genome of *P. aeruginosa* ATCC 27853 by IslandViewer [15] using SIGI-HMM [16] and IslandPath-DIMOB [17] algorithms. The lengths of these GIs range from 4055 bp to 36,677 bp with four GIs associated with prophages (Table 3, and below). Some genes in the remaining GIs were assigned to functional groups including metal resistance, virulence, regulatory proteins etc. (Table 3). Knowledge of the exact functions of these genes would require further investigations. Compared with PAO1, three GIs that are unique to *P. aeruginosa* ATCC 27853 contain a number of genes encoding monoxoygenase, dioxygenase and hydrolase, which are likely responsible for catabolism of aromatic compounds. Genes in these GIs were not annotated as they only displayed high similarity to certain genes present in a handful of draft genomes of *P. aeruginosa* strains that lack functional annotation.

### Prophages

**Fig. 3:**
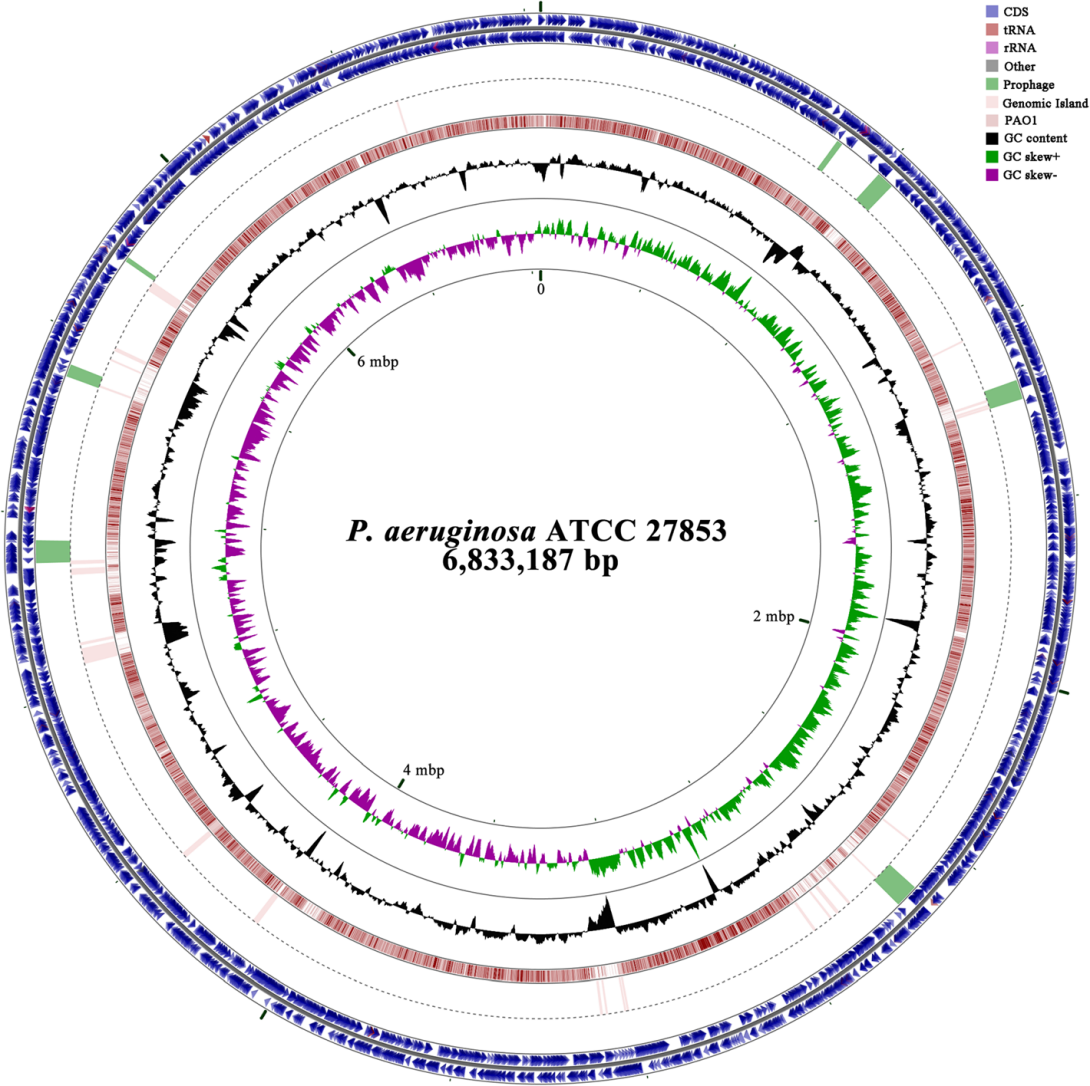
Circular genome map of *P. aeruginosa* ATCC 27853 showing the Genomic Islands (GIs) predicted by IslandViewer and prophages. From the outside: *circles* 1 and 2 (clockwise and counterclockwise) genes on the + and - strands, respectively; circles 3, prophages; 4, Genomic Islands; 5, PAO1 genes; 6, GC content; 7, GC skew. The scale in mbp is indicated on the innermost of the map

**Fig. 4:**
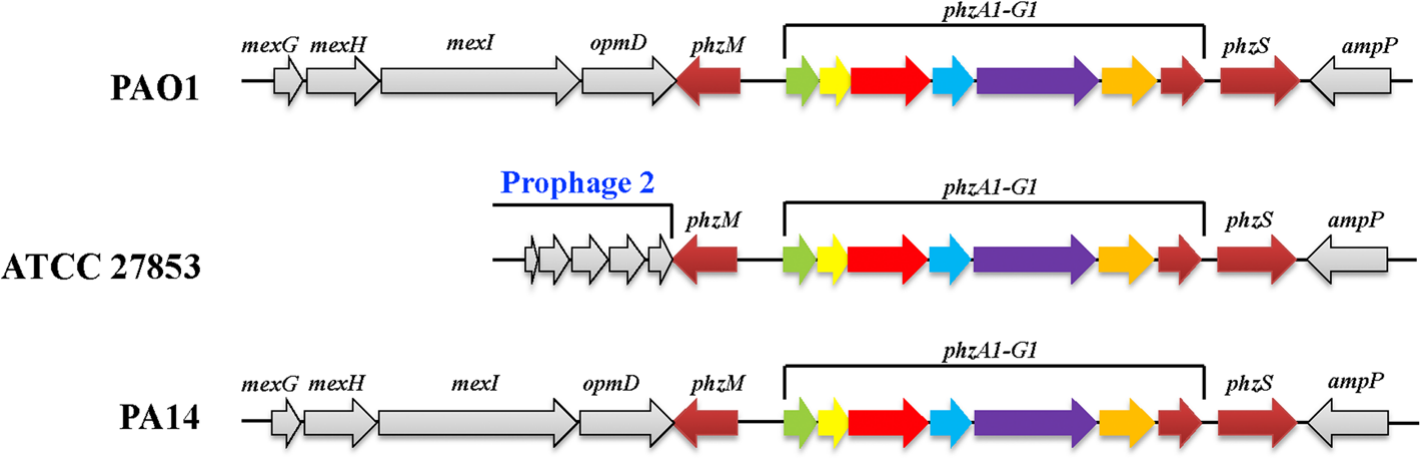
Comparison of the gene cluster of phenazine biosynthesis (Phz1) and its flanking regions in three strains of *P. aeruginosa*: ATCC 27853, PAO1 and PA14. Genomic location of the prophage 2 upstream of *phz1* gene cluster in ATCC 27853 is highlighted

Prophage prediction using Prophinder [18] and PHAST [19] revealed seven prophages in the genome of *P. aeruginosa* ATCC 27853. All these prophages were assigned as accessory genes and are designated as Prophage 1–7 (Table 4, Fig. 3). Prophage 1 which is closely related to phi CTX is located between genes encoding anthranilate synthase component I and component II. It is noteworthy that this prophage is observed in all available genomes of *P. aeruginosa* and its genomic location (between *trpE* and *trpG* genes) is also highly conserved, based on the PHASTER database [20]. The specific location of Prophage1 and its effect on the physiology of the *P. aeruginosa* host, particularly the antranilate biosynthesis, remain to be explored. Prophage 2 is 38,604 bp and harbors 50 open reading frames (ORFs). It is located between 797,729–836,333, upstream of the first phenazine biosynthesis gene cluster *phz1* (see below) (Fig. 4). This prophage does not interrupt any genes involved in phenazine biosynthesis (Fig. 4). Most ORFs in this prophage encode phage components such as phage head and tail, transposases and integrases (Fig. 3 and Additional file 1: Table S2). Besides these structural genes, one transcription factor which belongs to the DNA-binding IclR family could be annotated. A previous study showed that this prophage shares high similarity with prophage B3, a Mu-Like bacteriophage identified by Braid et al. (2004) [21]. Interestingly, prophage prediction in the complete genomes of *P. aeruginosa* revealed that this prophage exists in a few other *P. aeruginosa* strains such as NCGM2.S1, VRFPA04 and Carb01_63, but in different genome locations and with distinctive flanking regions (Additional file 1: Figure S1).

Prophage 3 is located at the genomic site of 1,337,276–1,379,950 with a size of 42,674 bp. Several genes that encode virulence associated proteins and transcriptional regulators were also identified, such as ACG06_06430 (gene locus tag in the genome of ATCC 27853 annotated by NCBI) which belongs to the LuxR family transcription factor that modulate quorum sensing [22]. Prophage 4 is the largest predicted prophages in ATCC 27853 genome and is composed of genes from different prophages such as phages ES18 and D3, indicating a complicated evolutionary history. In addition to typical phage components, other genes contained in the predicted prophages in the genome of *P. aeruginosa* ATCC 27853 include those of virulence factors and other functional genes, e.g. an adenylate kinase in Prophage 5.

### Phenazine biosynthesis

**Fig. 5:**
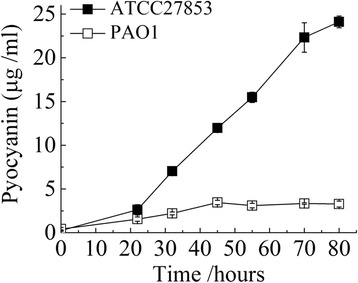
Measurements of pyocyanin in *P. aeruginosa* ATCC 27853 and PAO1 cultured in LB media

Phenazine compounds comprise an important class of secondary metabolites and virulence factors in *Pseudomonas* species. All phenazines contain a dibenzol annulated pyrazine ring represented by several structurally related compounds [23]. In most of the annotated *P. aeruginosa* genomes, two clusters of genes that encode phenazine biosynthetic pathways (Phz1 and Phz2) are present. The genes in the phenazine biosynthesis in ATCC 27853 and PAO1are highly similar (98.98 to 99.70% at nucleotide level). However, the *phz1* gene cluster in ATCC 27853 is preceded by Prophage 2 island (see above, ORFs with gene locus tags: ACG06_03785-ACG06_04040) (Fig. 4). On the other hand, the orthologous gene cluster of *phz1* in PAO1 (genes: PA4209-PA4217, gene locus tag in PAO1 from annotations by NCBI), is precededby a large fragment encompassing *opmD*, *mexI*, *mexH* and *mexG* genes (genes: PA4120-PA4208, Fig. 4) which are components of a Resistance Nodulation Division (RND) type efflux system and is proposed to pump the phenazine derivate 5-methylphenazine-1-carboxylate (5-Me-PCA) out of the cell [24]. These genes were absent in *P. aeruginosa* ATCC 27853. To examine whether this genomic difference affects phenazine production pattern, we measured the production of a major phenazine compound in *P. aeruginosa*, pyocyanin (PYO), in the two strains cultured in LB medium at room temperature. We observed a higher level of PYO in ATCC 27853 than in PAO1 at all time points examined (Fig. 5), suggesting that the different genomic architecture flanking the *phz1* gene cluster may indeed affect the PYO production in *P. aeruginosa* strains [25, 26].

### Virulence, surface-associated, and motility proteins

We compiled a database of 369 virulence genes based on a list of conserved virulence factors of *Pseudomonas* species with a primary focus on *P. aeruginosa* PAO1 and *P. aeruginosa* PA14 using the Virulence Factor Database (VFDB) [27] and the Victors Virulence Factors (PHIDIAS) (http://www.phidias.us/victors/index.php). Comparing ATCC 27853 genome against this database revealed that 254 of these virulence genes are also present in the genome of *P. aeruginosa* ATCC 27853 (Table 5). A class of virulence genes that are absent in *P. aeruginosa* ATCC 27853 include the *wbp* genes which encode the B-band lipopolysaccharide O antigen, with the exception of *wbpX*. B-band O-antigen of the lipopolysaccharide serotype O5 (such as that in PAO1) is important in conferring serum resistance in host pathogen interactions. Its presence or absence has also been shown to influence biofilm formation of the corresponding strain due to its capability to influence the hydrophilicity of cell surfaces and consequently the interaction of the cell with different surface materials and neighboring environment [28]. Absence of this system in *P. aeruginosa* ATCC 27853 probably indicates a defect in its defense mechanism against the host serum system and an altered biofilm formation capacity from that of the B^+^ strains such as PAO1.

**Fig. 6:**
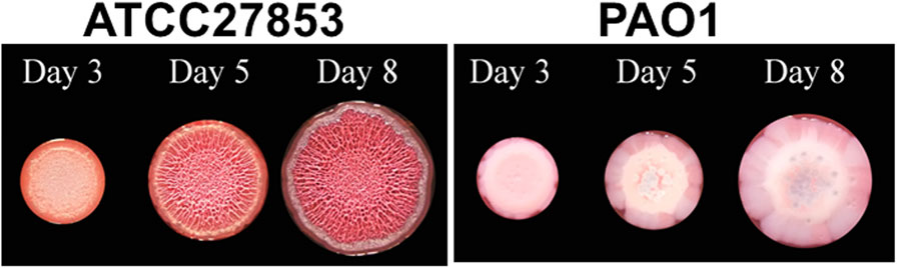
Colony morphology of *P. aeruginosa* ATCC 27853 and *P. aeruginosa* PAO1 cultured at 25 °C on LB agar plates supplemented with *Congo Red*

Interestingly, SNP distribution analysis in the genomes of PAO1 and ATCC 27853 revealed that a large number of non-synonymous variant sites present in the two strains are concentrated in the regions and genes that encode surface associated proteins, such as those that encode flagellar components, pyoverdine receptor, transporters, and type 4 pili (Additional file 1: Table S4 and Figure S2). These genomic differences combined suggest potentially different surface characteristics of ATCC 27853 when compared to PAO1. We therefore cultured the two strains on LB agar surface supplemented with Congo red and examined their capabilities to form colony biofilms [29]. A distinctive wrinkled colony morphology was observed in ATCC 27853 but not in PAO1 (Fig 6), suggesting a different surface pattern of ATCC 27853 compared with PAO1 and a strong capability of the strain to form biofilms. The stronger color of the ATCC 27853 biofilm compared to the biofilm of PAO1 on Congo red containing plate indicated a high level of exopolysaccharide matrix production in ATCC 27853, consistent with a stronger capability of the strain to form biofilm.

### Transcriptomes of *P. aeruginosa* ATCC 27853 and *P. aeruginosa* PAO1

The distinctive pattern of colony biofilms of ATCC 27853 and PAO1 shown above prompted us to investigate the functional genome of ATCC 27853 and compare it with that of PAO1 at that growth stage. We performed RNA-seq to obtain the complete transcriptomes of both strains cultured on LB agar surface at 25 °C, condition that is identical to that of colony biofilm formation described above. Cell cultures following 48 h incubation were harvested and RNA was extracted and sequenced as described in Materials and Methods. Statistical analysis including total reads number and bases sequenced, genome coverage, CDS coverage and mapping ratio for each sample from RNA-seq analyses are presented in supplementary data (Additional file 1: Table S5). To conduct a genome wide comparative gene expression analysis, orthologous genes between ATCC 27853 and PAO1 were first identified using reciprocal blastn and the ratio of their respective expression in the two strains was calculated by DESeq (Additional file 1: Table S3) [30].

**Fig. 7:**
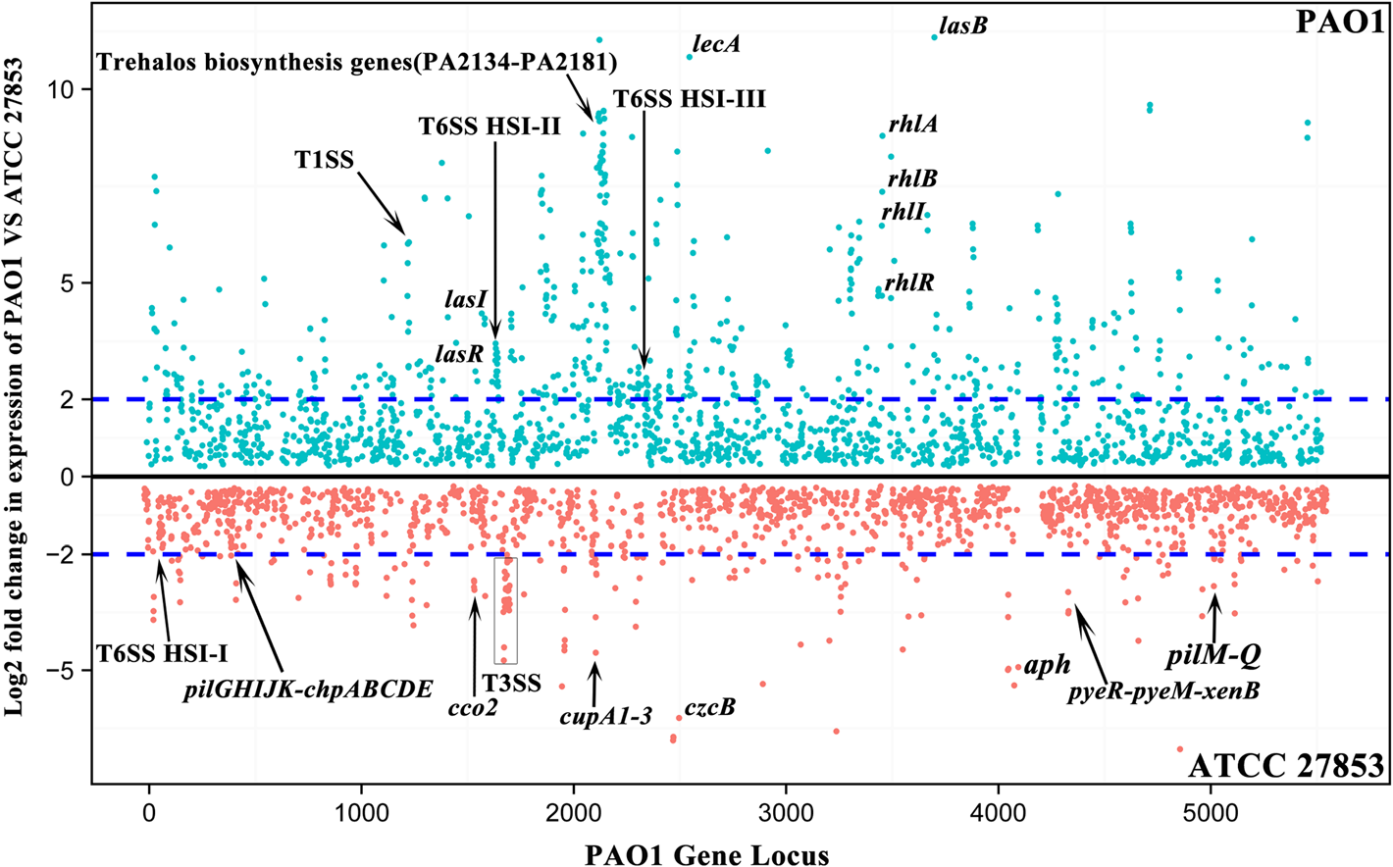
The genome wide transcriptomic profile of *P. aeruginosa* ATCC 27853 and PAO1. *Green dots* represent genes with higher relative expression level in PAO1 and *red dots* indicate genes with higher relative expression levels in ATCC 27853. The *blue dashed lines* represent log_2_-fold changes in expression. Selective genes and operons with distinctive expression patterns in the two strains are indicated

One hundred thirty seven genes with higher expression levels (log_2_ fold changes over 2) in ATCC 27853 than in *P. aeruginosa* PAO1 (Fig. 7, Additional file 1: Table S3) were identified. These include several classes of genes involved in biofilm formation, such as the type IV pili biogenesis gene cluster (*pilQPONM*: PA5040-PA5044) which is involved in the initiation of biofilms. Genes encoding twitching motility proteins, *pilGHIJK-chpABCDE* (PA0408-PA0417) were expressed at a higher level in ATCC 27853 than in PAO1 (Fig. 7, Additional file 1: Table S3). *pilABCDE* (PA4525-PA4528), *pilTU* (PA0395-PA0396) and *pilSR*-*yfiT*-*fimTU*-*pilVWXY1Y2E* (PA4546-PA4556) were also identified to display a slightly higher expression level in ATCC 27853 than in PAO1 (Additional file 1: Table S3). Expression of a proton motive force gene (pfm) (PA2950) that encodes a protein involved in energy metabolism critical for the rotation of flagellum in *P. aeruginosa* [31] was also higher in ATCC 27853 than in PAO1 (Fig. 7, Additional file 1: Table S3). Additionally, several other genes which are not directly involved in biofilm formation but have been reported to mediate the process were also found to be expressed at a higher level in ATCC 27853 than in PAO1, such as Chaperone-usher pathway (*cup*) A (PA2128-PA2132, *cupA1-A4*) encoding genes which were found to be required for adhesion to inert surfaces [32, 33], the *cbb3*-type cytochrome c oxidase *cco2* gene cluster (*ccoN2O2Q2P2*, PA1555-PA1557) which has been shown to promote biofilm formation under hypoxia through NO induction and its effect on cell elongation [34], as well as *pyeR* (PA4354) that encodes a non-classical ArsR family member of transcriptional regulators modulating biofilm formation in *P. aeruginosa* [35] (Fig. 7, Additional file 1: Table S3). All these genetic and transcriptional data support the distinct colony morphogenesis observed in ATCC 27853.

**Fig. 8:**
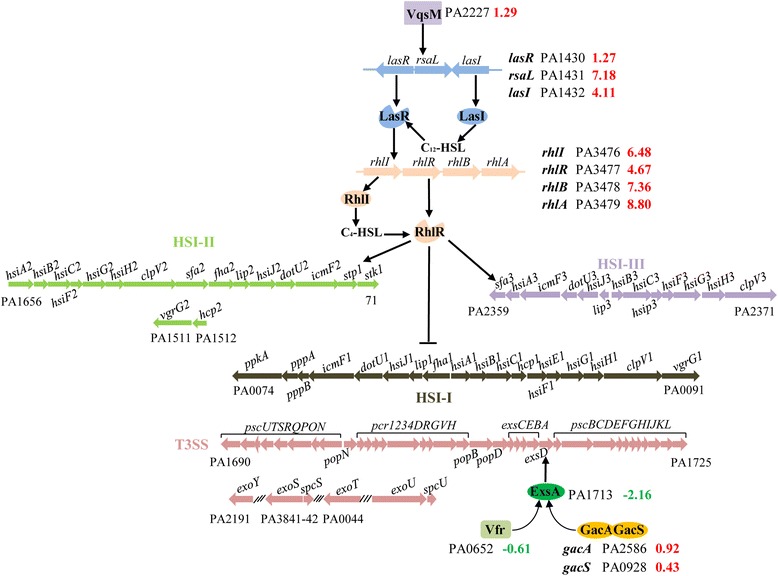
Differential expression of the genes involved in the type III and type VI secretion systems and their regulators in *P. aeruginosa* ATCC 27853 and PAO1. Gene locus tags in PAO1 are shown. Values following gene locus tags of regulators indicate Log2 gene expression changes in PAO1 relative to that in ATCC 27853 (*red* color indicates higher expression in PAO1 than in ATCC27853, *green* color indicates higher expression level in ATCC 27853 than in PAO1). The full list of genes displaying differential expression in the two strains and their values are provided in supplementary Additional file 1: Table S3

On the other hand, a much larger number (532 genes vs 137 as mentioned above) of genes with higher expression levels (log_2_ fold changes over 2) in *P. aeruginosa* PAO1 than in ATCC 27853 were observed (Fig. 7, Additional file 1: Table S3). Of particular prominence is a large fragment (PA2134-PA2181) of genes encoding trehalose biosynthesis. The homologous genes of this fragment in PA14 have been demonstrated to be involved in infection of plants [36]. Genes encoding several other virulence factors, such as *lecA* (encoding galactophilic lectin LecA) and *lasB* (encoding elastase LasB) were expressed at a higher level in PAO1 than in ATCC 27853 (Additional file 1: Table S3). It was also noticed that several transcriptional regulators which are quorum sensing genes mediating virulence factor production such as LasI, LasR, and RhlI and RhlR were also expressed at a higher level in PAO1 than in ATCC 27853 (Fig. 8).

An interesting observation is the expression patterns of the genes encoding various secretion systems in *P. aeruginosa* species. The components of type III secretion systems (T3SSs), such as genes in *psc*, *pcr* and *exs* gene clusters, display remarkably higher expression levels in ATCC 27853 than in PAO1 (Figs. 7 and 8), whereas those of the type I secretion system, namely T1SS, display a relatively higher expression level in PAO1 than in ATCC 27853. In the case of the type VI secretion system (T6SS) which includes three hemolysin co-regulated protein (Hcp) secretion islands HSI-I, II, III, while HSI-I was found to display a higher relative expression level in ATCC 27853 than in PAO1, that of HSI-II and III is opposite, i.e., they are expressed at higher level in PAO1 than in ATCC 27853 (Figs. 7 and 8).

## Discussion

### Morphogenesis in PAO1 and ATCC 27853

Surface characteristics play an important role in the morphogenesis of bacteria. *P. aeruginosa* is a well established model strain to study biofilms [37]. Outer membrane LPS and extracellular appendages, such as flagella, type IV pili and Cup fimbriae, are involved in the initial attachment of bacteria to a surface [38]. The present comparative genomic and transcriptomic study on *P. aeruginosa* ATCC 27853 and PAO1 revealed distinct genetic and expression pattern of surface associated proteins in ATCC 27853. Lacking of the B-band O-antigen (A^+^B^−^) has been reported to lead to an increased hydrophobicity of the cell surface and an enhanced adherence to polystyrene materials [39]. Increased expression of type IV pili biosynthesis genes and flagella motility genes also enhances bacterial adherence to various surfaces during the initiation of a biofilm. Our transcriptome analysis supports the expression patterns of these genes in ATCC 27853 which is consistent with the observed enhanced colony biofilm formation of the strain.

Three types of exopolysaccharides, alginate, Psl and Pel, play an important role in the biofilm maturation and development stage. Alginate has been proposed not to be a critical component of the extracellular polysaccharide matrix in nonmucoid *P. aeruginosa* strains [40]. The low expression levels of alginate biosynthesis genes in PAO1 and ATCC 27853 are consistent with the nonmucoid colony morphologies of the two strains. Previous studies demonstrated that Pel and Psl have distinct physical properties and functional roles during biofilm maturation and development [41]. The *pel* locus (referring to pellicle, a biofilm formed at the air-medium interface), containing the genes *pelA*-*G*, is responsible for synthesis of the glucose-rich component of the matrix, whereas the *psl* locus (*p*olysaccharide *s*ynthesis *l*ocus), containing the genes *pslA*-*O*, is responsible for the mannose- and galactose-rich component which forms a fiber-like matrix to enmesh bacterial communities [42]. Pel is required for close association of the two species in mixed-species microcolonies. In contrast, Psl is important for *P. aeruginosa* to form single-species biofilms. In the present study, expression of Pel biosynthesis genes were detected at a low level in both strains, however, a higher expression level of *psl* genes in ATCC 27853 compared to PAO1 was observed indicating a role of Psl in the development of ATCC 27853 colony biofilm. This result is also in agreement with a lower expression level of *amrZ* (PA3385) in ATCC 27853 than in PAO1, as the AmrZ transcriptional repressor controls switching between an alginate-producing mucoid state and a Pel-producing biofilm state through repression of *psl* genes [43, 44]. Another important signaling molecule which level in the cell correlates with the capability of the bacterium to form biofilms is the second messenger c-di-GMP. However, expression of several genes encoding diguanylate cyclase and phosphodiesterases which are involved in c-di-GMP production [45] was shown to be similar in PAO1 and ATCC 27853 in our comparative transcriptome analysis, suggesting that c-di-GMP did not play an important role in the distinctive colony biofilm formation observed in ATCC 27853 in comparison with that of PAO1.

Contribution of the phenazine compounds to the biofilm development of *P. aeruginosa* has also been reported [24, 46–48]. Recently, it was found that PYO can promote biofilm development of the bacterium by binding to extracellular DNA and enhancing the formation of extracellular matrix of biofilms [48]. Higher level of PYO production in ATCC 27853 than in PAO1 was observed in the present study. Thus, PYO may also contribute to the enhanced biofilm formation in ATCC 27853. The last step of PYO biosynthesis is the conversion of the zwitterionic intermediate 5-Me-PCA to the less charged PYO via hydroxylative decarboxylation. Interestingly, 5-Me-PCA, which is exported out of cells by the MexGHI-OmpD RND type efflux pump, was also shown to mediate the biofilm formation of *P. aeruginosa* in PA 14 [24]. It was proposed that export of 5-Me-PCA serves as a detoxification means in *P. aeruginosa*, likewise the conversion of this molecule to PYO which decreases the charge of the molecule and allows the transport of the product (PYO) across the membrane without the assistance of an efflux pump [24]. Indeed, PYO was shown not to be the substrate of the MexGHI-OmpD pump. The mexGHI-ompD system is present in both PAO1 and PA14, but is lacking in ATCC 27853. Yet, a higher level of PYO is observed in ATCC 27853 than in PAO1. This suggests that ATCC 27853 may contain other detoxification means allowing production of PYO in high level but minimizing the potential cytotoxicity of the intermediate 5-Me-PCA. Indeed, our genomic analysis revealed considerable differences of the two strains in terms of the numbers (122) of COGs. There are 71 unique COGs present in ATCC 27853 but are absent in PA14 and 51 COGs present in PA14 are lacking in ATCC 27853 (Fig. 2). These interesting observations warrant a comparative, molecular analysis of the PYO biosynthesis in ATCC 27853, PAO1, and PA14.

### Phylogenetic relations and accessory genes of *P. aeruginosa* ATCC 27853

In the phylogenetic tree constructed (Fig. 1), ATCC 27853 was shown to be extraordinarily closely related to three strains, *P. aeruginosa* T38079, *P. aeruginosa* F9670 and *P. aeruginosa* S86968. This phenomenon is interesting. Sequences of the three strains, T38079, F9670 and S86968, became available only very recently in the NCBI GenBank, and we included them in our phylogenetic analysis. However, this observation does not necessarily mean that these four strains are almost identical. This is because the SNPs utilized to construct the phylogenetic tree were extracted from the core genomic regions of all 59 strains which complete genome sequences are available. The SNPs do not cover the accessory genomes which are unique to each of the strains. Thus, the resulting relatedness of the strains in the phylogenetic tree does not reflect their associations at the complete genome level. Nevertheless, in the dataset we extracted, only 146 SNPs among these four strains were identified. Furthermore, the three strains and ATCC 27853 are assigned to the same multi-locus sequence type (MLST, https://pubmlst.org/paeruginosa/) and the same phylogenetic group based on NCBI GenBank, indicating very similar genomic contents of these four strains.

Core genome and accessory genes are two main components of the genomes of different *P. aeruginosa* strains [2]. Accessory genes are associated with genomic islands and islets that are attributed to diversification of strains within the species. This is termed as diversifying selection. Certain selective pressure might be responsible for the acquiring of these accessory genes and the resulting genome diversity among the different strains within the same species.

With the complete genome of *P. aeruginosa* ATCC 27853 on hand, its accessory genes were extensively examined in the current study. Within these accessory genes, the most prominent observation was the presence of seven prophages. Prophages contained in the genome of bacteria have been shown to play important roles in the physiology of the host bacterial species [49]. For example, two tandem defective phage (pyocin) islands on the *P. aeruginosa* PAO1 genome are the determinants of fluoroquinolone susceptibility of the strain [4]. Another study on *P. aeruginosa* LESB58 (Liverpool Epidemic Strain) demonstrated that the four prophages present in its genome could enhance competitiveness of the strain in a chronic rat lung infection model [5]. The abundance of prophages in the genome of ATCC 27853 implies the complexity and strong fitness potential of the strain. However, expression of these prophages was found to be low or non-detectable in the present study based on the transcriptome data (Fig. 7). This probably was due to the rich growth medium used in this study. Elucidating the functions of the genes within these prophages especially those encoding several transcriptional factors may help to disclose the potential roles of the prophages in the fitness of ATCC 27853 to the non-laboratory, harsh environments in nature and in animal hosts.

### Secretion systems

Secretion systems are important for the adaptation and pathogenesis of *P. aeruginosa* through dedicated secretion of specific exoproteins [50]. It has been shown that type III secretion systems (T3SSs) are correlated with acute infections in *P. aeruginosa*, while type VI secretion systems are often associated with chronic infections and biofilm formation of the species [47]. In the present study, genes encoding T3SS were found to be expressed at a remarkably higher level in ATCC 27853 than in PAO1 (Figs. 7 and 8). The genes encoding transcriptional activators of T3SS, e.g. *exsA* were also expressed at higher level in ATCC 27853 than in PAO1. Interestingly, a differential expression pattern of the three Hcp islands of T6SS was observed in these two strains, while HSI-II and HSI-III was expressed at a higher level in PAO1, HSI-I expression was higher in ATCC 27853 (Fig. 8). The three Hcp islands of *P. aeruginosa* have been assigned to different phylogenetic groups based on phylogenetic analysis, indicating a distinct evolutionary history of the three components [51]. This also suggests different roles of these three HSI islands during pathogenesis of *P. aeruginosa*. In addition, previous studies have demonstrated that the expression of these three Hcp islands of T6SS is mediated by different regulators [47]. LasR and RhlR positively regulate the expression of HSI-II and HSI-III gene clusters and LasR negatively regulates the HSI-I gene cluster in *P. aeruginosa* [47]. This is consistent with the higher expression level of LasR and RhlR in PAO1 compared with that in ATCC 27853 (Fig. 8). These observations indicate the complex expression patterns and functional roles of these secretion systems in the physiology and pathogenicity of different *P. aeruginosa* strains.

## Conclusions

In summary, several genomic features of *P. aeruginosa* ATCC 27853 were identified based on the complete genome sequence generated using Pacific Biosciences SMRT (PacBio) technology. Comparing with the genomes of the other two frequently used model strains *P. aeruginosa* PAO1 and PA14, three unique genomic islands were present in *P. aeruginosa* ATCC 27853 which contain genes possibly related to the metabolisms of aromatic compounds. Seven prophages are predicted including the prophage 2 which is located adjacent to the *phz1* phenazine biosynthesis gene clusters. Survey of virulence related genes revealed the lack of a gene cluster encoding the B-band O-antigen of LPS in *P. aeruginosa* ATCC 27853 which is important in evading of host immune responses and biofilm formation. Transcriptome analysis revealed differential gene expression of several groups of surface associated proteins and those involved in cellular redox metabolism, and the type I, III and VI secretion systems, confirming the different surface characteristics of ATCC 27853 from that of PAO1 and suggesting unique physiological and pathogenic potentials of ATCC 27853. These information provides genetic basis for the comprehensive understanding of the physiology, pathogenicity, and virulence of the strain.

## Methods

### Culture of bacterial cells and genomic DNA extraction

*P. aeruginosa* ATCC 27853 used in the present study was a gift obtained from Chinese University of Hong Kong (CUHK). It was cultivated in Luria-Bertani (LB) broth overnight with shaking (150 rpm) at 37 °C. Bacterial cells were harvested from 1 ml liquid culture via centrifugation at 10,000 rpm for 10 min. Genomic DNA of *P. aeruginosa* ATCC 27853 was extracted using QIAamp DNA Mini Kit (Qiagen, Hilden, Germany). The concentration and quality of genomic DNA was determined by NanoDrop and gel electrophoresis.

### Colony morphology assay

Congo red plates were prepared following the protocol described by Dietrich et al. with slight modifications [29]. Briefly, 1% tryptone and 1% agar were mixed with 40 μg/ml Congo red and 20 μg/ml Coomassie blue and poured on the square petri dish. 10 μl of overnight culture of *P. aeruginosa* inoculated from single colonies was spotted onto the square agar plates followed by incubation at 25 °C up to 9 days. Colony morphologies were recorded daily.

### Extraction and quantification of pyocyanin

Pyocyanin from liquid cultures harvested at different time point were extracted and measured following the protocol used by Recinos et al. and Apidianakis et al. with slight modification [26, 52]. Supernatant was collected after centrifugation at 13,000 rpm for 5 min and mixed with 0.6 volume of chloroform following vortex for 10 s twice. After centrifugation at 13,000 rpm for 5 min, blue layer at the bottom was transferred to a new tube and mixed with 0.5 volume of 0.2 M HCl with vortex for 10 s twice. 0.1 ml of the pink layer was transferred to a 96-well plate after 13,000 rpm for 5 min. Absorbance was determined at 510 nm.

### RNA preparation

Total RNA was extracted from triplicates of both ATCC 27853 and PAO1. 10 μl of overnight culture of *P. aeruginosa* PAO1 and ATCC 27853 in LB Broth inoculated from single colonies was spotted on the LB agar surface and incubated at 25 °C for 2 days. Cell patches were scraped from the plates and resuspended in 1 ml LB medium. 0.125 ml ice-cold phenol/ethanol stop solution (5:95, *v*/v, Ambion™ water saturated phenol at pH 6.6) was mixed with bacterial culture and placed on ice for 10 min to stop mRNA degradation. The mixture was subsequently centrifuged at 4800 rpm for 10 min at 4 °C. The supernatant was removed and cell pellet was stored at −80 °C for RNA extraction. RNA extraction was following the manufacture’s instructions using RNeasy Mini kit (Qiagen, Hilden, Germany). The quality of the extracted RNA has passed the Agilent Bioanalyzer analysis in Genome Research Centre of The University of Hong Kong (all RNA Integrity Number, RIN, are over 7). Stranded libraries for all RNA samples were constructed with Kapa Biosystems RNA library preparation chemistry in Georgia Genomics Facility at University of Georgia.

### Sequencing and de novo assembly

The whole genome sequencing of *P. aeruginosa* ATCC 27853 was performed using the PacBio RS II single-molecule, real-time sequencing system (SMRT) platform using 20 kb insert library and P6-C4 chemistry (Pacific Biosciences, Menlo Park, CA) by Macrogen(Korean). Raw SMRT reads were error corrected, de novo assembled the polished using the SMRT Analysis workflow [53] from Pacific Biosciences. The genome was checked for circularization by self-aligning the contig and inspecting the dotplot for sticky edges (dotplot was created in Geopard [54]). Circularization was carried out by trimming one end of the contig then collapsing using Minimus2 [55]. The genome of *P. aeruginosa* ATCC 27853 and transcriptomes of the two strains, PAO1 and *P. aeruginosa* ATCC 27853 were sequenced on the Illumina NextSeq platform (Illumina, San Diego, California, USA) using a run of 300 Cycles PE150 High Output Flow Cell in the Georgia Genomics Facility at the University of Georgia. DNA-seq raw reads from *P. aeruginosa* ATCC 27853 were aligned to the single PacBio contig and the Variant Call Format (VCF) file was generated with SAMtools [56].

### Genome annotations

Automated gene calling and annotation was carried out using the National Center for Biotechnology Information (NCBI)‘s Prokaryotic Genome Annotation Pipeline 2.0 (PGAP) [57]. We assessed and validated the annotation by comparing to that from the Rapid Annotations using Subsystems Technology (RAST) Server [58] as well as that from Prokka [59]. tRNA genes were predicted using tRNAscan-SE 1.3.1 [60] and rRNA genes using RNAmmer 1.2 [61]. Metabolic pathways were predicted in silico using KAAS [62]. Protein sequences of *P. aeruginosa* ATCC 27853 were BLAST-ed against the Clusters of Orthologous Groups (COG) database with an e-value score of 1e-5 [63].

### Prediction of prophage and genomic islands

Prophages in the genome of *P. aeruginosa* ATCC 27853 were predicted using the online softwares Prophinder with parameters (Scanning window size: 20,50,100,200,300; Minimum nb of CDS in prophages: 20; Minimum nb of ACLAME hits: 20; Blast Eval threshold: 1e-5; Minimum DR size: 10) [18] and PHAST [19]. IslandViewer was used with two methods including SIGI-HMM and IslandPath-DIMOB [15] to predict genomic islands (GIs). Hypothetic genes in prophages or GIs annotated by methods mentioned above were also blasted against the Pfam database constructed based on protein modules to improve annotations [64]. In addition, all available complete genomes of *P. aeruginosa* in Genbank were surveyed with PHAST to predict prophages [19].

### Virulence gene prediction

In *P. aeruginosa* PAO1, 273 virulence genes were identified based on a conserved list of 369 virulence genes in *Pseudomonas* species obtained from the Virulence Factor Database (VFDB) [27], Victors Virulence Factors (PHIDIAS) (http://www.phidias.us/victors/index.php), and curation by the *Pseudomonas* Genome Database (PseudoCAP) [65] with a primary focus on *P. aeruginosa* PAO1 and *P. aeruginosa* PA14. These 273 virulence proteins were blasted against all proteins in ATCC 27853 through BLASTp with 1e-5 e-value. Those without positive result of the blast search were recognized as absent in ATCC 27853. All the protein sequences of ATCC 27853 were also blasted against this conserved list of virulence genes with 1e-5 e-value.

### Comparative analysis of genomes

Four draft genomes of *P. aeruginosa* ATCC 27853 were recruited from Genbank (Table 1) [9–12] and compared with the complete genome obtained in the current study. 58 complete genomes of *P. aeruginosa* were also retrieved from Genbank and were compared with ATCC 27853 using *progressiveMauve* with default settings [66]. Proteins present exclusively in an individual strain and those shared between two or three strains based on Mauve and COG blast analysis were counted and represented in Venn diagrams generated by VennDiagram in *R*-platform [67]. For single nucleotide polymorphisms (SNPs) calling between PAO1 and ATCC 27853, VCF was first generated using Parsnp from Harvest tools [13]. VCF was annotated using SnpEff using PAO1 as reference genome [68].

**Table 1:** List of the genomic features of *P. aeruginosa* ATCC 27853 revealed from the complete genome (this study) and those of previous publications

Features	ATCC 27853	LCT-PA102	LCT-PA41	Boston 41,501	LCT-PA220
Number of scaffolds greater than or equal to 500 bp	1	124(296)	48(191)	1(10)	45(194)
Total length (bp)	6,833,187	6,887,913	6,887,679	6,819,384	6,746,593
% GC Content	66.12%	66.15%	66.16%	66.1%	66.17%
Genes	6366	6474	6476	6295	6464
rRNA	12	-	-	31	-
tRNA	66	54	47	66	54
Tandem repeats	215	-	186	-	210
Reference	This study	Fang et al. 2012 [9]	Liu et al. 2014 [10]	Minogue et al. 2014 [11]	Xu et al. 2014 [12]

“-” indicates that data is not available

**Table 2:** COG identified in *P. aeruginosa* ATCC 27853 but absent in PAO1

COG ID	Gene locus tag	Annotation
COG0213	ACG06_12750	Thymidine phosphorylase
COG0270	ACG06_12115 ACG06_23945	Site-specific DNA methylase
COG0338	ACG06_04040 ACG06_25705	Site-specific DNA methylase
COG0641	ACG06_22850	Arylsulfatase regulator (Fe-S oxidoreductase)
COG0798	ACG06_13015	Arsenite efflux pump ACR3 and related permeases
COG1205	ACG06_26950	Distinct helicase family with a unique C-terminal domain including a metal-binding cysteine cluster
COG1223	ACG06_23485	Predicted ATPase (AAA+ superfamily)
COG1479	ACG06_19220	Uncharacterized conserved protein
COG1541	ACG06_14110	Coenzyme F390 synthetase
COG1783	ACG06_12260	Phage terminase large subunit
COG2189	ACG06_23500	Adenine specific DNA methylase Mod
COG2253	ACG06_23380	Uncharacterized conserved protein
COG2372	ACG06_12630	Uncharacterized protein, homolog of Cu resistance protein CopC
COG2856	ACG06_29550	Predicted Zn peptidase
COG3383	ACG06_20450	Uncharacterized anaerobic dehydrogenase
COG3421	ACG06_23505	Uncharacterized protein conserved in bacteria
COG3440	ACG06_26970	Predicted restriction endonuclease
COG3464	ACG06_12825	Transposase and inactivated derivatives
COG3567	ACG06_12270	Uncharacterized protein conserved in bacteria
COG3575	ACG06_27620	Uncharacterized protein conserved in bacteria
COG3657	ACG06_25795	Uncharacterized protein conserved in bacteria
COG3723	ACG06_12140	Recombinational DNA repair protein (RecE pathway)
COG3762	ACG06_12760	Predicted membrane protein
COG4096	ACG06_22820	Type I site-specific restriction-modification system, R (restriction) subunit and related helicases
COG4245	ACG06_22780	Uncharacterized protein encoded in toxicity protection region of plasmid R478, contains von Willebrand factor (vWF) domain
COG4248	ACG06_22770	Uncharacterized protein with protein kinase and helix-hairpin-helix DNA-binding domains
COG4371	ACG06_15075 ACG06_15120 ACG06_15175 ACG06_15230	Predicted membrane protein
COG4373	ACG06_03925	Mu-like prophage FluMu protein gp28
COG4387	ACG06_03965	Mu-like prophage protein gp36
COG4570	ACG06_12235	Holliday junction resolvase
COG4938	ACG06_22795	Uncharacterized conserved protein
COG5005	ACG06_03940	Mu-like prophage protein gpG
COG5268	ACG06_18730	Type IV secretory pathway, TrbD component
COG5283	ACG06_23880	Phage-related tail protein
COG5484	ACG06_23765	Uncharacterized conserved protein
COG5492	ACG06_12325	Bacterial surface proteins containing Ig-like domains
COG5518	ACG06_23760	Bacteriophage capsid portal protein
COG5569	ACG06_12595	Uncharacterized conserved protein
COG5639	ACG06_18690	Uncharacterized conserved small protein

**Table 3:** List of genomic islands identified in *P. aeruginosa* ATCC 27853

Start	End	Size (bp)	Gene locus tag range	Mainly annotated genes
1,208,749	1,213,145	4396	ACG06_05790- ACG06_05800	-
1,357,527	1,363,441	5914	ACG06_06510- ACG06_06545	Prophage 3
1,369,295	1,374,450	5155	ACG06_06585 ACG06_06595	Prophage 3
1,375,007	1,379,950	4943	ACG06_06610- ACG06_06645	Prophage 3
2,433,632	2,438,325	4693	ACG06_11700- ACG06_11730	General secretion pathway protein
2,550,405	2,554,460	4055	ACG06_12335- ACG06_12355	Prophage 4
2,635,133	2,641,374	6241	ACG06_12645- ACG06_12670	Heavy metal, cooper response
2,668,513	2,677,091	8578	ACG06_12800- ACG06_12845	Mercuric resistance
2,690,535	2,695,956	5421	ACG06_12920- ACG06_12960	-
2,736,058	2,742,733	6675	ACG06_13180- ACG06_13200	Virulence
3,210,856	3,216,077	5221	ACG06_14975- ACG06_15000	Hydrolase
3,217,225	3,221,802	4577	ACG06_15010- ACG06_15040	Hypothetical protein
3,260,302	3,265,059	4757	ACG06_15285- ACG06_15305	Antibiotics biosynthesis
3,271,888	3,278,016	6128	ACG06_15330- ACG06_15350	Monooxygenase and hydrolase
4,118,826	4,132,842	14,016	ACG06_19215 ACG06_19245	Integrase and dehydrogenase
4,345,306	4,358,424	13,118	ACG06_20250- ACG06_20300	-
4,855,376	4,892,053	36,677	ACG06_22680- ACG06_22805	TetR family Transcriptional regulator
4,899,363	4,906,116	6753	ACG06_22830- ACG06_22850	Hypothetical protein
5,064,552	5,079,564	15,012	ACG06_23610- ACG06_23680	PFGI-1-like_cluster_1
5,089,064	5,097,821	8757	ACG06_23720- ACG06_23755	Peptidase and Thioredoxin
5,506,547	5,511,513	4966	ACG06_25640- ACG06_25670	Prophage 6
5,573,404	5,577,851	4447	ACG06_26025- ACG06_26055	Mercuric resistance
5,597,700	5,606,740	9040	ACG06_26180- ACG06_26220	Transcriptional regulator
5,757,464	5,780,636	23,172	ACG06_26905- ACG06_26970	Bacterial regulatory proteins, AsnC family
6,493,061	6,497,532	4471	ACG06_30145- ACG06_30150	Hypothetical protein

**Table 4:** List of prophages identified in *P. aeruginosa* ATCC 27853

Prophage ID	Score	Normalized	Coordinate range	Length	Win size	Close phage
Prophage 1	15.3525	0.9031	683,173–696,044	12,871	20	phi CTX
Prophage 2	63.7992	1.3292	797,729–836,333	38,604	50	B3
Prophage 3	44.6107	0.6759	1,337,276–1,379,950	42,674	100	F10/lambda
Prophage 4	53.0771	0.8042	2,508,898–2,560,402	51,504	100	D3/ES18
Prophage 5	47.7643	0.7582	5,093,820–5,142,761	48,941	100	phi CTX
Prophage 6	1.6659	0.0490	5,504,457–5,531,885	27,428	50	B3
Prophage 7	13.3825	0.8364	5,783,641–5,794,256	10,615	20	Pf1

Score. It is a significance score from Prophinder, more reliable prediction with higher score

Normalised. Each significant score is normalized based on its number of CDS

Win size. Prophinder runs with different window sizes to screen the genome. Here is reported with which window size the prophage was detected

**Table 5:** List of virulence genes present in PAO1 but is absent or with low identity in *P. aeruginosa* ATCC 27853

PAO1	ATCC 27853	Identity on protein level (%)	Annotation
PA1092	ACG06_21330	63.04	Flagellin type B
PA1093	ACG06_21325	41.48	Hypothetical protein/flaG protein
PA1094	ACG06_21320	43.07	Flagellar capping protein FliD
PA1095	ACG06_21310/ACG06_21315	66.40/39.67	Flagellar protein FliS
PA1096	ACG06_21305	46.94	Hypothetical protein
PA2397	ACG06_14125	64.55	PvdE, pyoverdine biosynthesis protein
PA2398	ACG06_14120	28.37	Ferripyoverdine receptor
PA2399	N/A	N/A	PvdD, pyoverdine biosynthetase D
PA2400	N/A	N/A	PvdJ, pyoverdine biosynthetic process
PA2402	ACG06_14105	69.40	Probable non-ribosomal peptide synthetase
PA2424	ACG06_13995/ACG06_14110	99.61/49.19	Probable non-ribosomal peptide synthetase/PvdL
PA2427	ACG06_13980	33.33	Hypothetical protein
PA3142	N/A	N/A	Integrase
PA3143	N/A	N/A	Transposase
PA3144	N/A	N/A	Transposase with Helix-turn-helix Hin domain
PA3145	ACG06_09210	63.85	Glycosyltransferase WbpL
PA3146	ACG06_09205	37.78	Probable NAD-dependent epimerase/dehydratase WbpK
PA3147	ACG06_09195	24.10	Probable glycosyl transferase WbpJ
PA3148	N/A	N/A	UDP-N-acetylglucosamine 2-epimerase WbpI
PA3149	N/A	N/A	Probable glycosyltransferase WbpH
PA3150	N/A	N/A	LPS biosynthesis protein WbpG
PA3153	N/A	N/A	O-antigen translocase
PA3154	N/A	N/A	B-band O-antigen polymerase
PA3156	N/A	N/A	UDP-2-acetamido-3-amino-2,3-dideoxy-d-glucuronic acid N-acetyltransferase, WbpD
PA3157	N/A	N/A	Probable acetyltransferase, WbpC
PA3158	N/A	N/A	UDP-2-acetamido-2-deoxy-d-glucuronic acid 3-dehydrogenase, WbpB
PA3159	N/A	N/A	UDP-N-acetyl-d-glucosamine 6-Dehydrogenase,WbpA
PA3160	ACG06_09160	54.95	O-antigen chain length regulator, Wzz
PA3487	N/A	N/A	Tle5,Secreted Factors (toxins, enzymes, alginate)
PA3498	ACG06_28265	44.79	Probable oxidoreductase
PA4150	N/A	N/A	Probable dehydrogenase E1 component
PA4175	N/A	N/A	Protease IV
PA4197	N/A	N/A	BfiS, Two-component System
PA4525	ACG06_25540	41.67	Type 4 fimbrial precursor PilA
PA4527	ACG06_25550	N/A	Frameshift type 4 fimbrial biogenesis protein PilC (putative pseudogene)

### Phylogenetic analyses

The phylogenetic analysis was performed to validate the phylogenetic position of *P. aeruginosa* ATCC 27853. Parsnp from Harvest tools [13] was employed with default settings to collect single nucleotide polymorphisms (SNPs) from all currently available complete genomes of *P. aeruginosa* and 269,561 SNPs were submitted for phylogenetic analysis with a maximum likelihood (ML) criterion in MEGA [14]. Parameters for this analysis included: Tamura-Nei substitution model, Gamma Distributed Rates among sites, Nearst-Neighbor-Interchange (NNI) ML Heuristic method for tree inference options, using automatically generated initial tree with NJ method, and 100 times bootstrap test.

### RNA-seq quality processing

We performed quality control (QC) on the raw Illumina RNA-Seq data using BBduk2 (BBMap short read aligner, http://sourceforge.net/projects/bbmap). Reads were culled based on a minimum average quality of 20 over a window of 7 bp. Low quality read edges were trimmed and reads containing more than two ambiguous bases were removed. Finally, read pairs were trimmed evenly and a minimum length of 60 bp was enforced.

### RNA-seq read mapping

QC reads were mapped to their respective reference genomes in two stages. First, QC reads were aligned using BWA-MEM with default parameters [69]. The second round of read mapping was conducted using Stampy with the output from BWA-MEM (with Stampy’s --bamkeepgoodreads -M options) [70]. SAMtools and BamTools were used for format conversions, statistics, and quality assessment and control [53, 71]. IGV tools were also used to visually inspect mapping quality to ensure its accuracy [72].

### Fragment counts and statistics

Fragment (our RNA-Seq data are stranded) counting per genomic features (genes) was performed using featureCounts [73]. Reads that mapped with MAPQ scores below 10 were removed. Enforcing a MAPQ score below 10 also excludes multi-mapped reads albeit the percentage of this category is low (data not shown). Multi-mapping was determined using default parameters. Read pairs were checked for proper pairing as well as the proper insert size. Counting was performed for each gene based on its locus_tag. Read counts were used as input for DESeq analysis [30]. Genes with mean normalized expression <50 reads in all samples were considered as transcriptional noise and filtered out from the analysis. In DESeq, fold changes (log_2_(fold-change) ≥ 2or ≤2) for each expression gene and *p*-value < 0.05 [cut-off at 5% false discovery rate (FDR)] was employed as threshold for the statistics analysis.

## Additional file

Additional file 1: Table S1. A list of complete genomes of *Pseudomonas aeruginosa* employed in the present study. **Table S2.** Annotations of ORFs in Prophage 2 predicted in *P. aeruginosa* ATCC 27853. **Table S3.** Table S3 Differentially expressed genes in *P. aeruginosa* ATCC 2853 and PAO1 revealed by DESeq of the RNA-seq data (see supplemented excel file). **Table S4** Top 50 ranked genes with numbers of non-synonymous variants between *Pseudomonas aeruginosa* ATCC 2853 and PAO1 with function description. **Table S5** RNA-seq statistics and coverage after quality filtering for PAO1 and ATCC 27853. **Fig. S1.** Location of prophage B3 in four *P. aeruginosa* genomes: ATCC 27853, *P. aeruginosa* NCGM2.S1, *P. aeruginosa* VRFPA04 and *P. aeruginosa* Carb01_63. **Fig. S2** Distribution of nucleotide change numbers in the genomes of *P. aeruginosa* ATCC 27853 and PAO1. (ZIP 718 kb)

## Abbreviations

COG: Clusters of orthologous group
GI: Genomic islands
Hcp: Hemolysin co-regulated protein
HGAP: Hierarchical Genome Assembly Process
HSI: Hcp secretion island
NCBI: The National Center for Biotechnology Information
ORFs: Open reading frames
RAST: Rapid Annotations using Subsystems Technology
SMRT: Single Molecule Real-Time
T3SS: Type III secretion systems
T6SS: Type VI secretion system
