# Supplementary material for: Comparative genome and transcriptome analysis reveals distinctive surface characteristics and unique physiological potentials of Pseudomonas aeruginosa ATCC 27853
Source: BMC Genomics. 2017 Jun 12;18:459. doi: 10.1186/s12864-017-3842-z (PMC5467263; doi:10.1186/s12864-017-3842-z)
Supplement: Additional file 1: Table S1. — A list of complete genomes of Pseudomonas aeruginosa employed in the present study. Table S2. Annotations of ORFs in Prophage 2 predicted in P. aeruginosa ATCC 27853. Table S3. Table S3 Differentially expressed genes in P. aeruginosa ATCC 2853 and PAO1 revealed by DESeq of the RNA-seq data (see supplemented excel file). Table S4 Top 50 ranked genes with numbers of non-synonymous variants between Pseudomonas aeruginosa ATCC 2853 and PAO1 with function description. Table S5 RNA-seq statistics and coverage after quality filtering for PAO1 and ATCC 27853. Fig. S1. Location of prophage B3 in four P. aeruginosa genomes: ATCC 27853, P. aeruginosa NCGM2.S1, P. aeruginosa VRFPA04 and P. aeruginosa Carb01_63. Fig. S2 Distribution of nucleotide change numbers in the genomes of P. aeruginosa ATCC 27853 and PAO1. (ZIP 718 kb) [file 12864_2017_3842_MOESM1_ESM.zip › Suppl_revised.docx]

Table S1 A list of complete genomes of *Pseudomonas aeruginosa* employed in the present study.

| Strain Name | Source/Description | BioSample | BioProject | Size (Mb) | GC% | Genes | Proteins | Release Date | Ref. |
| --- | --- | --- | --- | --- | --- | --- | --- | --- | --- |
| PAO1 | A wound in Melbourne, Australia | SAMN02603714 | PRJNA331 | 6.26 | 66.6 | 5697 | 5572 | 2000/5/16 | Stover et al., 2000 |
| PACS2 | Cystic fibrosis patient | SAMN02471994 | PRJNA16851 | 6.49 | 66.3 | 5990 | 5904 | 2006/6/5 | N/A |
| UCBPP-PA14 | A clinical isolate from a human burn patient | SAMN02603591 | PRJNA386 | 6.54 | 66.3 | 5976 | 5873 | 2006/10/6 | Lee et al., 2006 |
| PA7 | A clinical isolate | SAMN02603435 | PRJNA16720 | 6.59 | 66.4 | 6070 | 5932 | 2007/7/5 | Roy et al., 2010 |
| LESB58 | Liverpool epidemic strain (LES) | SAMEA1705916 | PRJEA31101 | 6.60 | 66.3 | 6125 | 6005 | 2008/12/24 | Winstanley et al., 2008 |
| 19BR | Polymixin B adaptation strain | SAMN02471401 | PRJNA70773 | 6.74 | 66.1 | 6256 | 6129 | 2011/8/17 | Boyle et al., 2012 |
| 213BR | Polymixin B adaptation strain | SAMN02471402 | PRJNA70775 | 6.72 | 66.1 | 6231 | 6110 | 2011/8/17 | Boyle et al., 2013 |
| M18 | Sweet melon rhizosphere | SAMN02603849 | PRJNA61423 | 6.33 | 66.5 | 5820 | 5717 | 2011/9/19 | Wu et al., 2011 |
| NCGM2.S1 | Highly multidrug-resistant strain from Japan | - | PRJDA73453 | 6.76 | 66.1 | 6274 | 6140 | 2011/9/30 | Miyoshi-Akiyama et al., 2011 |
| DK2 | A cystic fibrosis patient | SAMN02603895 | PRJNA73815 | 6.40 | 66.3 | 5919 | 5788 | 2012/6/20 | Rau et al., 2012 |
| B136-33 | Infant with community-acquired diarrhea | SAMN02603669 | PRJNA185969 | 6.42 | 66.4 | 5877 | 5771 | 2013/4/9 | N/A |
| RP73 | Persistent Cystic Fibrosis | SAMN02603771 | PRJNA206088 | 6.34 | 66.5 | 5849 | 5724 | 2013/6/24 | Jeukens et al., 2013 |
| PA1 | A Patient with a Respiratory Tract Infection | SAMN02603191 | PRJNA185335 | 6.50 | 66.4 | 6014 | 5910 | 2013/11/12 | Lu et al., 2015 |
| PA1R | N/A | SAMN02603192 | PRJNA185336 | 6.31 | 66.3 | 5862 | 5752 | 2013/11/12 | N/A |
| MTB-1 | Environmental strain | SAMN02389526 | PRJNA225944 | 6.58 | 66.2 | 6062 | 5953 | 2013/12/5 | Ohtsubo et al., 2014 |
| LES431 | Liverpool epidemic strain (LES) from the UK and Canada | SAMN02641592 | PRJNA222518 | 6.55 | 66.3 | 6052 | 5925 | 2013/12/17 | N/A |
| SCV20265 | Highly adherent *P.aeruginosa* small-colony variant SCV20265 | SAMN02415141 | PRJNA229445 | 6.73 | 66.3 | 6248 | 6131 | 2013/12/18 | Eckweiler et al., 2014 |
| YL84 | Compost, found to be a chitinase-producing quorum-sensing bacterium | SAMN02641599 | PRJNA234476 | 6.43 | 66.4 | 5908 | 5785 | 2014/1/30 | Chan et al., 2014 |
| VRFPA04 | Human corneal button from India | SAMN02472098 | PRJNA219238 | 6.82 | 66.5 | 6291 | 5841 | 2014/6/27 | N/A |
| NCGM 1900 | Urinary catheter | SAMD00018700 | PRJDB2972 | 6.81 | 66 | 6335 | 6206 | 2014/8/1 | N/A |
| NCGM 1984 | Urinary catheter | SAMD00019082 | PRJDB3032 | 6.85 | 66.0 | 6380 | 6257 | 2014/9/18 | N/A |
| F22031 | Pubic bone, infected cancer patients | SAMN02673269 | PRJNA237986 | 6.60 | 66.2 | 6077 | 5968 | 2015/1/9 | N/A |
| FRD1 | Sputum of a cystic fibrosis patient | SAMN02732380 | PRJNA245215 | 6.71 | 66.1 | 6182 | 5871 | 2015/1/29 | N/A |
| NCGM257 | Midstream urine | SAMD00020552 | PRJDB3186 | 7.09 | 65.9 | 6687 | 6546 | 2015/2/25 | N/A |
| NCTC10332 | Derivant from NCTC_3000 | SAMEA2479570 | PRJEB6403 | 6.32 | 66.5 | 5798 | 5627 | 2015/3/22 | N/A |
| PA8380 | Human gut | SAMD00027370 | PRJDB3600 | 6.61 | 66.2 | 6114 | 6004 | 2015/4/22 |  |
| Carb01 63 | Human-associated habitat | SAMN03389320 | PRJNA281807 | 7.50 | 65.6 | 7060 | 6901 | 2015/4/29 | N/A |
| DSM 50071 | *P. aeruginosa* (Schroeter 1872) Migula 1900 (DSM 50071T) | SAMN03761153 | PRJNA285827 | 6.32 | 66.5 | 5801 | 5709 | 2015/7/2 | Nakano et al., 2015 |
| F9676 | A diseased rice sample in 2009 | SAMN03852256 | PRJNA289296 | 6.37 | 66.5 | 5825 | 5721 | 2015/7/13 | N/A |
| PA1RG | A phage-resistant mutant of PA1 strain | SAMN04074798 | PRJNA295473 | 6.50 | 66.3 | 6021 | 5917 | 2015/9/17 | Li et al., 2016 |
| VA-134 | Skin wound of burn human patient | SAMN04284690 | PRJNA302926 | 6.40 | 66.4 | 5858 | 5725 | 2015/11/24 | Miller et al., 2016 |
| IOMTU 133 | Urinary catheter | SAMD00042491 | PRJDB4304 | 6. 90 | 66.0 | 6324 | 6197 | 2015/11/26 |  |
| Cu1510 | Industrial waste water | SAMN04102969 | PRJNA296749 | 6.12 | 66.7 | 5629 | 5285 | 2015/12/10 | Yan et al., 2016 |
| AES-1R | Sputum of a 14 month old infant with cystic fibrosis | SAMN02471202 | PRJNA64619 | 6.34 | 66.5 | 5877 | 5762 | 2015/12/22 | Naughton et al., 2011 |
| 12-4-4(59) | Blood culture of burn human patient | SAMN04351367 | PRJNA306518 | 6.43 | 66.3 | 5912 | 5802 | 2015/12/24 | Karna et al., 2016 |
| S86968 | Clinical isolate | SAMN02894350 | PRJNA253624 | 6.95 | 66.0 | 6437 | 6279 | 2016/1/15 | N/A |
| T38079 | Clinical isolate | SAMN02894349 | PRJNA253624 | 6.81 | 66.1 | 6251 | 6055 | 2016/1/15 | N/A |
| T52373 | Clinical isolate | SAMN02894348 | PRJNA253624 | 6.32 | 66.5 | 5749 | 5563 | 2016/1/15 | N/A |
| T63266 | Clinical isolate | SAMN02894347 | PRJNA253624 | 6.46 | 66.3 | 5960 | 5800 | 2016/1/15 | N/A |
| W16407 | Clinical isolate | SAMN02894346 | PRJNA253624 | 6.83 | 65.9 | 6285 | 6099 | 2016/1/15 | N/A |
| W36662 | Clinical isolate | SAMN02894345 | PRJNA253624 | 6.80 | 66.2 | 6290 | 6158 | 2016/1/15 | N/A |
| W45909 | Clinical isolate | SAMN02894344 | PRJNA253624 | 6.81 | 66.2 | 6288 | 6145 | 2016/1/15 | N/A |
| W60856 | Clinical isolate | SAMN02894343 | PRJNA253624 | 6.91 | 66.2 | 6380 | 6252 | 2016/1/15 | N/A |
| F23197 | Clinical isolate | SAMN02894358 | PRJNA253624 | 6.54 | 66.2 | 5953 | 5826 | 2016/1/15 | N/A |
| F30658 | Clinical isolate | SAMN02894357 | PRJNA253624 | 7.27 | 65.8 | 6715 | 6542 | 2016/1/15 | N/A |
| H5708 | Clinical isolate | SAMN02894355 | PRJNA253624 | 6.35 | 66.5 | 5827 | 5722 | 2016/1/15 | N/A |
| H27930 | Clinical isolate | SAMN02894354 | PRJNA253624 | 6.60 | 66.2 | 6042 | 5881 | 2016/1/15 | N/A |
| H47921 | Clinical isolate | SAMN02894353 | PRJNA253624 | 6.84 | 66.1 | 6322 | 6166 | 2016/1/15 | N/A |
| M1608 | Clinical isolate | SAMN02894352 | PRJNA253624 | 6.48 | 66.0 | 5955 | 5807 | 2016/1/15 | N/A |
| M37351 | Clinical isolate | SAMN02894351 | PRJNA253624 | 6.90 | 66.0 | 6389 | 6239 | 2016/1/15 | N/A |
| F63912 | Clinical isolate | SAMN02894356 | PRJNA253624 | 6.64 | 66.2 | 6196 | 6065 | 2016/1/15 | N/A |
| USDA-ARS-USMARC-41639 | Clinical isolate | SAMN04158502 | PRJNA281531 | 6.36 | 66.4 | 5838 | 5735 | 2016/1/20 | N/A |
| DHS01 | Multidrug-resistant clinical isolate | SAMN02383553 | PRJNA224149 | 7.06 | 65.8 | 6651 | 6524 | 2016/1/25 | Valot et al., 2014 |
| X78812 | Clinical isolate | SAMN02894342 | PRJNA253624 | 6.37 | 66.4 | 5823 | 5703 | 2016/1/29 | N/A |
| F9670 | Clinical isolate | SAMN02894359 | PRJNA253624 | 6.79 | 66.1 | 6237 | 5906 | 2016/1/29 | N/A |
| N17-1 | Aflatoxin B1 degradation strain | SAMN03145716 | PRJNA265138 | 6.37 | 66.4 | 5856 | 5763 | 2016/4/4 | N/A |
| PAO1 Orsay | PAO1 variants, resistant to bacteriophages | SAMEA3485223 | PRJEB9838 | 6.28 | 66.5 | 5766 | 5678 | 2015/9/8 | N/A |

N/A: not available.

Table S2 Annotations of ORFs in Prophage 2 predicted in *Pseudomonas aeruginosa* ATCC 27853.

| Gene_tag | Coordinates | Length | ACLAME hit | ACLAME function(s) | Annotations |
| --- | --- | --- | --- | --- | --- |
| ACG06_03795 | 797729 - 798295 | 188 | protein:vir:99840 - Eval: 2.00E-65 |  | bacteriophage protein |
| ACG06_03800 | 798282 - 798749 | 155 | protein:vir:99867 - Eval: 1.00E-58 |  | hypothetical protein |
| ACG06_03805 | 798749 - 798940 | 63 | protein:vir:79251 - Eval: 1.00E-16 | phi:0000326  - phage function unknown | hypothetical protein |
| ACG06_03810 | 798942 - 799631 | 229 | protein:vir:99835 - Eval: 1.00E-122 |  | hypothetical protein |
| ACG06_03815 | 799633 - 800256 | 207 | protein:vir:99836 - Eval: 1.00E-106 |  | sulfate transporter |
| ACG06_03820 | 800249 - 800449 | 66 | protein:vir:99885 - Eval: 2.00E-23 |  | bacteriophage protein |
| ACG06_03825 | 800442 - 800975 | 177 | protein:vir:99219 - Eval: 1.00E-24 | phi:0000326  - phage function unknown | hypothetical protein |
| ACG06_03830 | 800965 - 801645 | 226 | protein:vir:4398 - Eval: 7.00E-23 |  | hypothetical protein |
| ACG06_03835 | 801645 - 801929 | 94 | protein:vir:99891 - Eval: 4.00E-32 |  | hypothetical protein |
| ACG06_03840 | 801926 - 802267 | 113 | protein:vir:99878 - Eval: 2.00E-45 | phi:0000326  - phage function unknown | bacteriophage protein |
| ACG06_03845 | 802269 - 803435 | 388 | [protein:vir:99889 - Eval: 1.00E-148](http://aclame.ulb.ac.be/perl/Aclame/Genomes/prot_view.cgi?view=prot&id=protein:vir:99889) |  | transposase |
| ACG06_03850 | 803435 - 805219 | 594 | [protein:vir:99858 - Eval: 0.00E+00](http://aclame.ulb.ac.be/perl/Aclame/Genomes/prot_view.cgi?view=prot&id=protein:vir:99858) |  | integrase |
| ACG06_03855 | 805223 - 806197 | 324 | [protein:vir:99855 - Eval: 1.00E-21](http://aclame.ulb.ac.be/perl/Aclame/Genomes/prot_view.cgi?view=prot&id=protein:vir:99855) |  | hypothetical protein |
| ACG06_03860 | 806207 - 806521 | 104 | protein:vir:99842 - Eval: 7.00E-03 |  | hypothetical protein |
| ACG06_03865 | 806518 - 806778 | 86 | - |  | hypothetical protein |
| ACG06_03870 | 806771 - 807259 | 162 | [protein:vir:99843 - Eval: 1.00E-62](http://aclame.ulb.ac.be/perl/Aclame/Genomes/prot_view.cgi?view=prot&id=protein:vir:99843) |  | hypothetical protein |
| ACG06_03875 | 807387 - 807878 | 163 | - |  | hypothetical protein |
| ACG06_03880 | 807892 - 808122 | 76 | [protein:vir:99884 - Eval: 2.00E-12](http://aclame.ulb.ac.be/perl/Aclame/Genomes/prot_view.cgi?view=prot&id=protein:vir:99884) |  | phage-related DNA-binding protein |
| ACG06_03890 | 808584 - 809087 | 167 | [protein:vir:99886 - Eval: 2.00E-08](http://aclame.ulb.ac.be/perl/Aclame/Genomes/prot_view.cgi?view=prot&id=protein:vir:99886) |  | hypothetical protein |
| ACG06_03895 | 809239 - 809535 | 98 | [protein:vir:99854 - Eval: 4.00E-27](http://aclame.ulb.ac.be/perl/Aclame/Genomes/prot_view.cgi?view=prot&id=protein:vir:99854) |  | membrane protein |
| ACG06_03900 | 809693 - 810322 | 209 | [protein:vir:99856 - Eval: 6.00E-87](http://aclame.ulb.ac.be/perl/Aclame/Genomes/prot_view.cgi?view=prot&id=protein:vir:99856) |  | lytic murein transglycosylase |
| ACG06_03905 | 810524 - 811147 | 207 | protein:vir:99882 - Eval: 8.00E-59 |  | lysis protein |
| ACG06_03910 | 811147 - 811467 | 106 | - |  | bacteriophage protein |
| ACG06_03915 | 811464 - 811766 | 100 | [protein:vir:1983 - Eval: 8.00E-11](http://aclame.ulb.ac.be/perl/Aclame/Genomes/prot_view.cgi?view=prot&id=protein:vir:1983) |  | hypothetical protein |
| ACG06_03920 | 811769 - 812317 | 182 | [protein:vir:99873 - Eval: 1.00E-10](http://aclame.ulb.ac.be/perl/Aclame/Genomes/prot_view.cgi?view=prot&id=protein:vir:99873) | phi:0000074  - phage terminase small subunit | small terminase subunit |
| ACG06_03925 | 812319 - 813992 | 557 | [protein:vir:1985 - Eval: 0.00E+00](http://aclame.ulb.ac.be/perl/Aclame/Genomes/prot_view.cgi?view=prot&id=protein:vir:1985) | phi:0000073  - phage terminase large subunit | Mu-like prophage FluMu protein gp28 |
| ACG06_03930 | 813986 - 815560 | 524 | [protein:vir:103860 - Eval: 1.00E-119](http://aclame.ulb.ac.be/perl/Aclame/Genomes/prot_view.cgi?view=prot&id=protein:vir:103860) | phi:0000068  - phage portal protein | Mu-like prophage FluMu protein gp29 |
| ACG06_03935 | 815550 - 816788 | 412 | [protein:vir:99846 - Eval: 2.00E-62](http://aclame.ulb.ac.be/perl/Aclame/Genomes/prot_view.cgi?view=prot&id=protein:vir:99846) | phi:0000010  - phage head/capsid assembly | bacteriophage protein |
| ACG06_03940 | 816790 - 817365 | 191 | [protein:vir:99833 - Eval: 3.00E-54](http://aclame.ulb.ac.be/perl/Aclame/Genomes/prot_view.cgi?view=prot&id=protein:vir:99833) | phi:0000010  - phage head/capsid assembly | bacteriophage protein |
| ACG06_03945 | 817576 - 818685 | 369 | [protein:vir:103838 - Eval: 1.00E-59](http://aclame.ulb.ac.be/perl/Aclame/Genomes/prot_view.cgi?view=prot&id=protein:vir:103838) | phi:0000017  - phage prohead/capsid assembly | bacteriophage protein |
| ACG06_03950 | 818691 - 819095 | 134 | [protein:vir:103872 - Eval: 2.00E-18](http://aclame.ulb.ac.be/perl/Aclame/Genomes/prot_view.cgi?view=prot&id=protein:vir:103872) |  | bacteriophage protein |
| ACG06_03955 | 819110 - 820006 | 298 | [protein:vir:99228 - Eval: 3.00E-73](http://aclame.ulb.ac.be/perl/Aclame/Genomes/prot_view.cgi?view=prot&id=protein:vir:99228) | phi:0000066  - phage head/capsid major protein | Mu-like prophage major head subunit gpT |
| ACG06_03960 | 820238 - 820453 | 71 | [protein:vir:103835 - Eval: 6.00E-12](http://aclame.ulb.ac.be/perl/Aclame/Genomes/prot_view.cgi?view=prot&id=protein:vir:103835) | phi:0000326  - phage function unknown | hypothetical protein |
| ACG06_03965 | 820456 - 820971 | 171 | [protein:vir:99848 - Eval: 1.00E-32](http://aclame.ulb.ac.be/perl/Aclame/Genomes/prot_view.cgi?view=prot&id=protein:vir:99848) | phi:0000326  - phage function unknown | bacteriophage protein |
| ACG06_03970 | 820968 - 821420 | 150 | [protein:vir:99874 - Eval: 2.00E-31](http://aclame.ulb.ac.be/perl/Aclame/Genomes/prot_view.cgi?view=prot&id=protein:vir:99874) | phi:0000326  - phage function unknown | bacteriophage protein |
| ACG06_03975 | 821417 - 821620 | 67 |  |  | bacteriophage protein |
| ACG06_03980 | 821627 - 822367 | 246 | [protein:vir:99839 - Eval: 5.00E-69](http://aclame.ulb.ac.be/perl/Aclame/Genomes/prot_view.cgi?view=prot&id=protein:vir:99839) | phi:0000326  - phage function unknown | hypothetical protein |
| ACG06_03985 | 822370 - 822852 | 160 | [protein:vir:99838 - Eval: 4.00E-18](http://aclame.ulb.ac.be/perl/Aclame/Genomes/prot_view.cgi?view=prot&id=protein:vir:99838) | phi:0000326  - phage function unknown | bacteriophage protein |
| ACG06_03990 | 823151 - 826747 | 1213 | [protein:vir:99863 - Eval: 0.00E+00](http://aclame.ulb.ac.be/perl/Aclame/Genomes/prot_view.cgi?view=prot&id=protein:vir:99863) | phi:0000086  - phage tail tape measure protein | tail length tape measure protein |
| ACG06_03995 | 826747 - 827703 | 318 | [protein:vir:99862 - Eval: 1.00E-174](http://aclame.ulb.ac.be/perl/Aclame/Genomes/prot_view.cgi?view=prot&id=protein:vir:99862) | phi:0000326  - phage function unknown | hypothetical protein |
| ACG06_04000 | 827706 - 828629 | 307 | [protein:vir:103887 - Eval: 1.00E-162](http://aclame.ulb.ac.be/perl/Aclame/Genomes/prot_view.cgi?view=prot&id=protein:vir:103887) | phi:0000326  - phage function unknown | hypothetical protein |
| ACG06_04005 | 828632 - 830338 | 568 | [protein:vir:99844 - Eval: 0.00E+00](http://aclame.ulb.ac.be/perl/Aclame/Genomes/prot_view.cgi?view=prot&id=protein:vir:99844) |  | hypothetical protein |
| ACG06_04010 | 830325 - 831143 | 272 | [protein:vir:99865 - Eval: 1.00E-154](http://aclame.ulb.ac.be/perl/Aclame/Genomes/prot_view.cgi?view=prot&id=protein:vir:99865) | phi:0000015  - phage tail assembly | hypothetical protein |
| ACG06_04015 | 831153 - 831383 | 76 | [protein:vir:99864 - Eval: 5.00E-35](http://aclame.ulb.ac.be/perl/Aclame/Genomes/prot_view.cgi?view=prot&id=protein:vir:99864) | phi:0000174  - capsid tail | tail assembly protein |
| ACG06_04020 | 831389 - 831610 | 73 |  |  | hypothetical protein |
| ACG06_04025 | 831597 - 833807 | 736 | protein:vir:99834 - Eval: 0.00E+00 | phi:0000174  - capsid tail | bacteriophage protein |
| ACG06_04030 | 833804 - 834952 | 382 | [protein:vir:79213 - Eval: 1.00E-128](http://aclame.ulb.ac.be/perl/Aclame/Genomes/prot_view.cgi?view=prot&id=protein:vir:79213) | phi:0000175  - capsid tail fiber | hypothetical protein |
| ACG06_04035 | 834949 - 835239 | 96 | protein:vir:79211 - Eval: 2.00E-34 |  | hypothetical protein |
| ACG06_04040 | 835539 - 836333 | 264 | protein:vir:99841 - Eval: 1.00E-145 | go:0009008  - DNA-methyltransferase activity | restriction endonuclease subunit M |

Note: ACLAME stands for the Database of phage, plasmid, and transposons functional mobiles.

Table S3 Differentially expressed genes in *Pseudomonas aeruginosa* ATCC 27853 and PAO1 revealed by DEseq of the RNA-seq data with normalized abundance for each gene in both strains (see supplemented excel file).

Table S4 Top 50 ranked genes with numbers of non-synonymous variants between *Pseudomonas aeruginosa* ATCC 2853 and PAO1 with function description.

| Gene locus tag in PAO1 | Number of Non-synonymous variant | Number of Synonymous variant | Number of obtained Stop codon | Function |
| --- | --- | --- | --- | --- |
| PA1094 | 276 | 115 | 2 | Flagellar capping protein FliD |
| PA2397 | 256 | 182 | 7 | Pyoverdine biosynthesis protein PvdE |
| PA0041 | 200 | 152 | 2 | Hemagglutinin |
| PA1087 | 194 | 131 | 3 | Flagellar hook-associated protein FlgL |
| PA0726 | 189 | 116 | 3 | Hypothetical protein |
| PA0620 | 153 | 144 | 1 | Bacteriophage protein |
| PA1091 | 132 | 93 | 1 | Flagellar glycosyl transferase, FgtA |
| PA3145 | 119 | 151 | 3 | Glycosyltransferase WbpL |
| PA1086 | 118 | 156 | 0 | Flagellar hook-associated protein FlgK |
| PA2690 | 116 | 45 | 0 | Transposase |
| PA4554 | 116 | 135 | 2 | Type 4 fimbrial biogenesis protein PilY1 |
| PA1092 | 104 | 72 | 1 | Flagellin type B |
| PA0104 | 103 | 46 | 1 | Hypothetical protein |
| PA0595 | 94 | 211 | 3 | Organic solvent tolerance protein OstA |
| PA2386 | 93 | 131 | 0 | L-ornithine N5-oxygenase |
| PA4526 | 88 | 113 | 0 | Type 4 fimbrial biogenesis protein PilB |
| PA2392 | 87 | 155 | 2 | Protein PvdP |
| PA0470 | 86 | 129 | 0 | Ferrichrome receptor FiuA |
| PA2403 | 85 | 93 | 2 | Hypothetical protein |
| PA2388 | 72 | 93 | 0 | Protein FpvR |
| PA5088 | 67 | 112 | 0 | Hypothetical protein |
| PA1096 | 63 | 28 | 0 | Hypothetical protein |
| PA1093 | 62 | 44 | 1 | Hypothetical protein |
| PA0621 | 53 | 41 | 3 | Hypothetical protein |
| PA1150 | 49 | 32 | 1 | Pyocin S2 |
| PA1095 | 48 | 33 | 0 | Flagellar protein FliS |
| PA2396 | 46 | 97 | 2 | Pyoverdine synthetase F |
| PA2402 | 46 | 61 | 0 | Peptide synthase |
| PA2387 | 44 | 60 | 1 | RNA polymerase sigma factor |
| PA2462 | 38 | 73 | 0 | Hypothetical protein |
| PA2393 | 36 | 40 | 0 | Dipeptidase |
| PA5089 | 35 | 40 | 1 | Hypothetical protein |
| PA1874 | 33 | 63 | 0 | Hypothetical protein |
| PA2400 | 33 | 96 | 0 | Protein PvdJ |
| PA0958 | 30 | 66 | 0 | Porin |
| PA1271 | 30 | 19 | 0 | TonB-dependent receptor |
| PA4503 | 30 | 85 | 0 | ABC transporter permease |
| PA0992 | 25 | 38 | 0 | Fimbrial subunit CupC1 |
| PA1153 | 25 | 12 | 0 | Hypothetical protein |
| PA4525 | 23 | 26 | 1 | Type 4 fimbrial PilA |
| PA3141 | 22 | 42 | 1 | Nucleotide sugar epimerase/dehydratase WbpM |
| PA5040 | 22 | 29 | 1 | Type 4 fimbrial biogenesis outer membrane protein PilQ |
| PA2302 | 19 | 23 | 0 | Protein AmbE |
| PA2424 | 17 | 54 | 0 | Peptide synthase |
| PA4501 | 17 | 48 | 0 | Glycine-glutamate dipeptide porin OpdP |
| PA1267 | 16 | 22 | 0 | Hypothetical protein |
| PA0690 | 15 | 64 | 0 | Hypothetical protein |
| PA2404 | 14 | 14 | 0 | Hypothetical protein |
| PA3294 | 14 | 16 | 0 | Hypothetical protein |
| PA4974 | 14 | 23 | 0 | Probable outer membrane protein precursor OpmH |

Table S5 RNA-seq statistics and coverage after quality filtering for PAO1 and ATCC 27853.

| Strain | Replicate | # Reads ^1^ | # Bases ^2^ | Genome Coverage ^3^ | CDS Coverage ^4^ | Mapping ratio (%) |
| --- | --- | --- | --- | --- | --- | --- |
| PAO1 | A | 37073468 | 4514517492 | 720 | 807 | 99 |
|  | B | 39425456 | 4773943308 | 762 | 854 | 99 |
|  | C | 37390702 | 4551295985 | 726 | 814 | 99 |
| ATCC 27853 | A | 34438130 | 4235262557 | 619 | 704 | 91 |
|  | B | 33719044 | 4116359102 | 602 | 684 | 94 |
|  | C | 32232286 | 3939542947 | 576 | 655 | 95 |

Notes: 1. Total pair-end reads; 2. Total base pair count is based on both pair end reads; 3. Based on genome size: ATCC 27853 = 6,833,187 bp and PAO1 = 6,264,404 bp; 4. Based on Total CDS size: ATCC 27853 = 6,011,247 bp and PA1O = 5,587,732 bp.


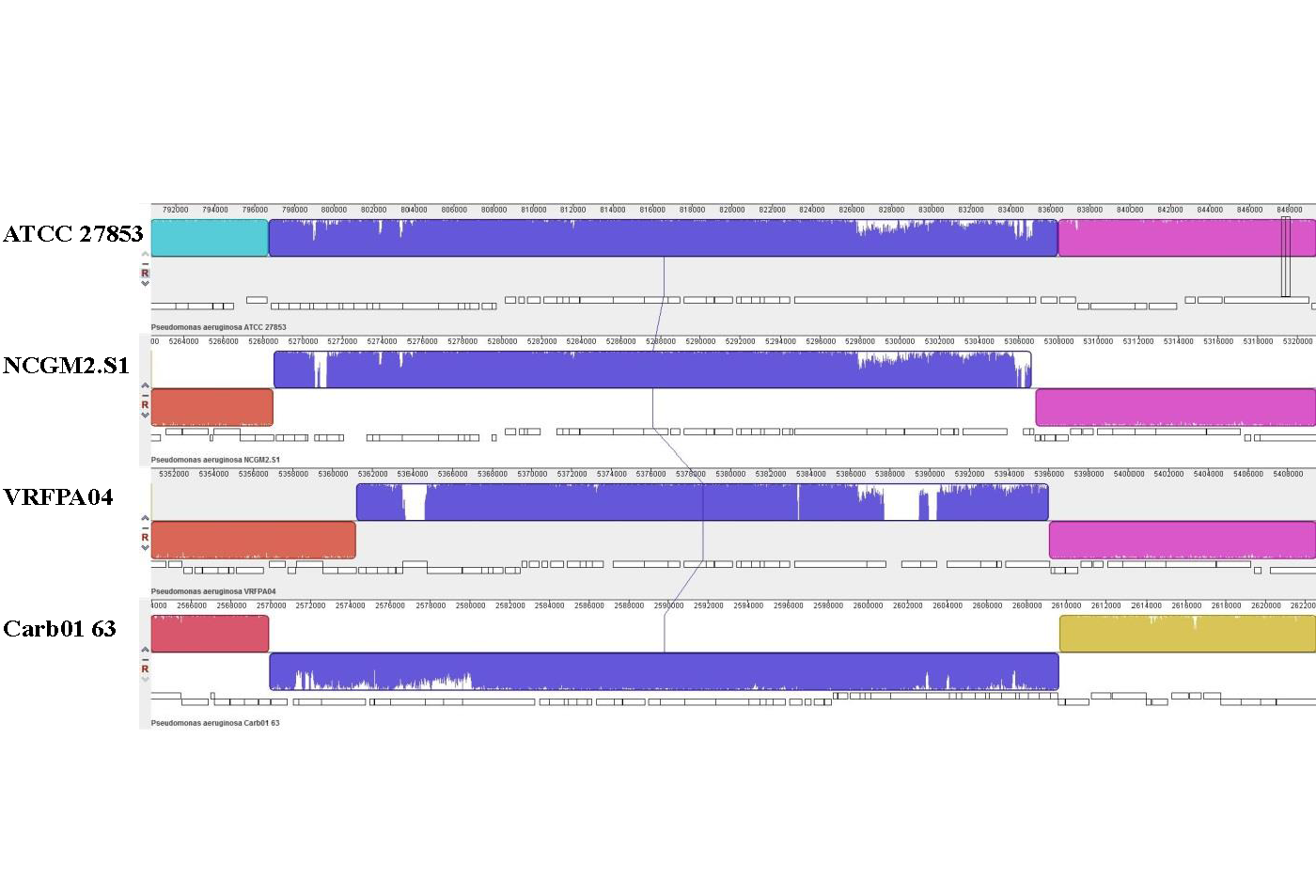


Fig. S1 Location of prophage B3 in four *P. aeruginosa* genomes: ATCC 27853, *P. aeruginosa* NCGM2.S1, *P. aeruginosa* VRFPA04 and *P. aeruginosa* Carb01 63. The violet domain in each of the genomes represents the prophage B3. Homologous gene clusters are indicated in the same color.


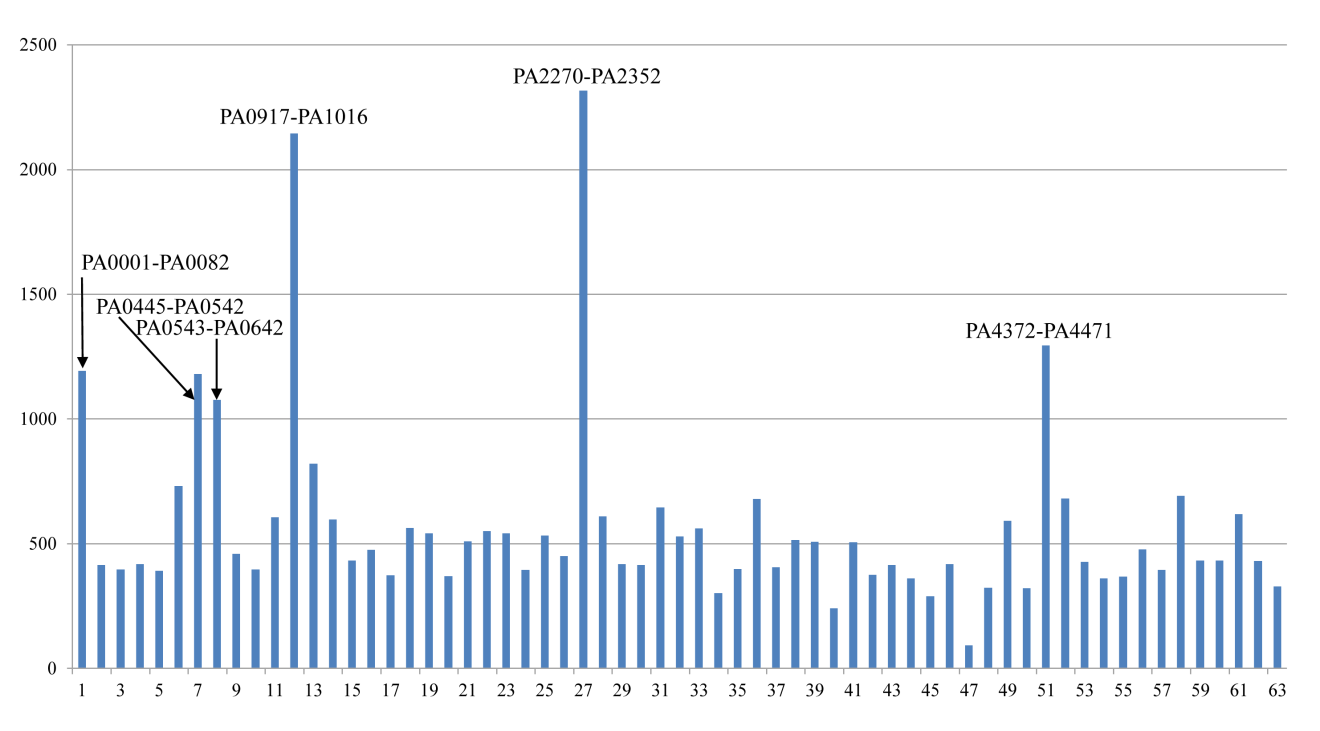
Fig. S2 Distribution of nucleotide change numbers in the genomes of *P. aeruginosa* ATCC 27853 and PAO1. X-axis represents the genome section scaled by per 100kb using PAO1 as reference and Y-axis stands for the numbers of nucleotide changes in each section of genome. Gene locus tags in 6 sections with over 1000 nucleotide changes are shown.

Scripts used in the Materials and Methods section:

BBduk2: *bbduk2.sh qtrim=rl trimq=20 minlength=60 tbo=t tpe=t minavgquality=20 maxns=2 maqb=7 ktrim=l ktrim=r ref=adapters.fa*;

featureCounts: *featureCounts -R -M -Q 10 -p -P -s 2 -t gene -g locus_tag –largestOverlap;*
